# Network-informed deconvolution of bulk immune gene co-expression reveals single-cell programs and spatial organization

**DOI:** 10.3389/fimmu.2026.1843602

**Published:** 2026-09-01

**Authors:** Yiming Li, Yujie You, Ruixian Chen, Peiqing Wang, Yiming Zhang, Le Zhang, Senyi Deng

**Affiliations:** 1 Thoracic Department, Institute of Thoracic Oncology, West China Hospital, West China Medical School, Sichuan University, Chengdu, China; 2 School of Computer Science and Engineering, Sichuan University of Science and Engineering, Yibin, China; 3 Breast Center, West China Hospital, Sichuan University, Chengdu, China; 4 College of Computer Science, Sichuan University, Chengdu, China

**Keywords:** Bayesian network inference, bulk RNA sequencing, LUAD (lung adenocarcinoma), network biomarkers, PBMC (peripheral blood mononuclear cells), single cell transcriptomics

## Abstract

**Introduction:**

Single-cell RNA sequencing (scRNA-seq) has opened unprecedented possibilities to explore the complexity of the immune system. However, existing methods primarily rely on expression-based clustering analysis, which lacks mechanistic explanations for immune cell states and encounters challenges in integrating multi-scale data.

**Methods:**

We developed a network-informed deconvolution framework that constructs Bayesian network-derived regulatory structures using immune-related genes from context-matched bulk RNA-seq datasets. Network markers were extracted from these structures and projected onto peripheral blood mononuclear cell (PBMC) and lung adenocarcinoma (LUAD) scRNA-seq datasets to identify network biomarkers and define immune cell states. Spatial transcriptomic analysis was further used to evaluate the spatial coherence of network-defined cell states. The scRNA-seq and spatial transcriptomic datasets analyzed in this study were generated from prospectively collected samples by our team, while context-matched bulk RNA-seq cohorts were used to derive population-level immune gene network structures.

**Results:**

The framework identified structure-defined immune subpopulations in both PBMC and LUAD datasets and revealed functional heterogeneity across multiple immune lineages. Spatial transcriptomic analysis further showed that network-associated immune clusters exhibited closer spatial proximity than non-associated clusters, supporting the spatial coherence of network-defined cell states.

**Discussion:**

This framework provides a network-informed representation for immune cell subpopulation identification and functional characterization. By linking bulk immune gene co-expression, single-cell programs, and spatial organization, this approach offers an additional perspective for understanding immune dynamics in both normal and pathological states and may provide an analytical basis for more precise immunotherapy-related studies.

## 1 Introduction

With the rapid advancement of single cell RNA sequencing (scRNA-seq) technologies, researchers can now profile complex cellular systems at unprecedented resolution (1, 2). In the immune system in particular, scRNA-seq has enabled comprehensive characterization of lineage architecture, activation states, and functional heterogeneity under both homeostatic and pathological conditions (3–5). By applying dimensionality reduction and unsupervised clustering to highly variable gene expression matrices, scRNA-seq reveals previously unrecognized cell subpopulations and provides critical insights into the organization of immune landscapes at the tissue level (6–8). However, prevailing single cell analysis workflows remain largely driven by expression-based clustering and annotation using predefined marker genes (7, 9). Although these approaches perform well on standard datasets, they fundamentally rely on label-based strategies that lack mechanistic interpretability. Because clustering is based primarily on transcriptional similarity rather than shared regulatory logic, current methods often fail to resolve the underlying drivers of cellular identity or capture transitional and functionally related cell subtypes (10, 11). These limitations motivate the development of a framework inferring cell subtypes through network-informed structural analysis and advancing the biological interpretability and robustness of functional heterogeneity modeling.

In contrast to scRNA-seq, bulk RNA-seq lacks single-cell resolution but offers several technical advantages for regulatory network modeling, including larger sample sizes, higher sequencing depth, and lower technical noise (12). In recent years, Bayesian networks, which infer probabilistic dependencies and putative directional relationships among variables, have been widely applied to multi-omics integration, transcriptional regulatory modeling, and disease mechanism studies (13–18). Compared to conventional correlation-based methods such as WGCNA, Bayesian networks provide richer structural information, capturing core regulatory modules and backbone pathways with superior generalizability and biological interpretability (19–23). Taken together, bulk and single-cell modalities are complementary for Bayesian regulatory-network inference: bulk affords power and mature algorithms but cannot decipher cell type–specific regulation, whereas scRNA-seq excels at heterogeneity analysis but faces noise and analytical complexity. Current research has not yet fully integrated these two advantages, resulting in cell state classification in single-cell analysis still relying on empirical expression features rather than mechanistic models. Therefore, integrating these advantages—and projecting bulk-inferred regulatory structure onto single-cell resolution—remains a key challenge in contemporary single-cell research.

Spatial transcriptomics further provides a complementary layer for evaluating whether transcriptionally defined immune cell states are spatially organized within tissue architecture (24, 25). Although dissociation-based scRNA-seq captures cellular heterogeneity at high resolution, it loses information regarding local cellular neighborhoods and positional relationships among immune, tumor, and stromal cells. Therefore, spatial information can help assess whether cell states identified from single-cell transcriptomes show coherent organization within the tumor microenvironment (26, 27).

To address this challenge, we propose a framework that constructs a Bayesian network-derived regulatory structure from bulk RNA-seq data and systematically maps its structure onto single cell transcriptomes to identify immune cell functional states with network-level interpretability (28). We first curated a gene set based on immune-related terms from the Gene Ontology biological process category and employed it to build up a Bayesian regulatory network, thereby ensuring that the network topology can reflect immune specific regulatory modules. From this network, we systematically extracted high confidence regulatory subgraphs composed of 2 or 3 genes, referred to as network markers, which serve as structure-driven functional units (28–30). These markers were then projected onto single cell expression matrices, where their co-expression across cells enabled the identification of subpopulations exhibiting consistent regulatory structure. Network markers with high co-expression frequency within a cluster were subsequently aggregated into network biomarkers, thus defining cell subtypes that are jointly supported by regulatory topology, expression coherence, and functional annotation.

Rather than replacing conventional expression-based clustering methods such as Seurat, our framework provides a complementary network-informed layer for immune cell state interpretation. First, network markers, defined as two- or three-gene directed subgraphs, represent coordinated multi-gene structures rather than isolated transcripts. Second, network biomarkers aggregate recurrently co-expressed network markers within cell clusters, thereby linking expression coherence with bulk-derived network architecture. Third, by combining structure-guided marker usage with downstream functional annotation, the approach provides an additional perspective for resolving functionally distinct immune subsets in complex tissues.

We systematically evaluated this framework in both peripheral blood immune cells and lung adenocarcinoma samples. Our results demonstrate that the network biomarker system can identify discrete functional clusters across multiple immune lineages, with strong agreement observed across expression profiles, GO enrichment patterns, and spatial distribution. Furthermore, spatial transcriptomic analysis (31) revealed that cell populations defined by strongly connected network biomarker pairs tend to colocalize in tissue space, suggesting that regulatory structure may contribute to spatial organization.

Taken together, we present a structure-informed framework for single-cell state identification that enables the projection of bulk-inferred regulatory networks onto single-cell resolution. This approach establishes a generalizable methodology for network-informed decomposition of immune cell heterogeneity, cross-scale deconvolution of immune gene co-expression, and network-based biomarker construction. Recent advances in organoid-based disease modeling and studies of cancer cell biophysical properties further highlight that tumor heterogeneity can be investigated across diverse model systems and biological dimensions (32, 33). Beyond peripheral blood and lung adenocarcinoma, the framework is broadly applicable to a wide range of tissue contexts and cellular systems, offering a universal strategy for mapping transcriptional states to regulatory logic.

## 2 Results

### 2.1 Network-informed framework for immune cell state identification and spatial coherence analysis

**Figure 1:**
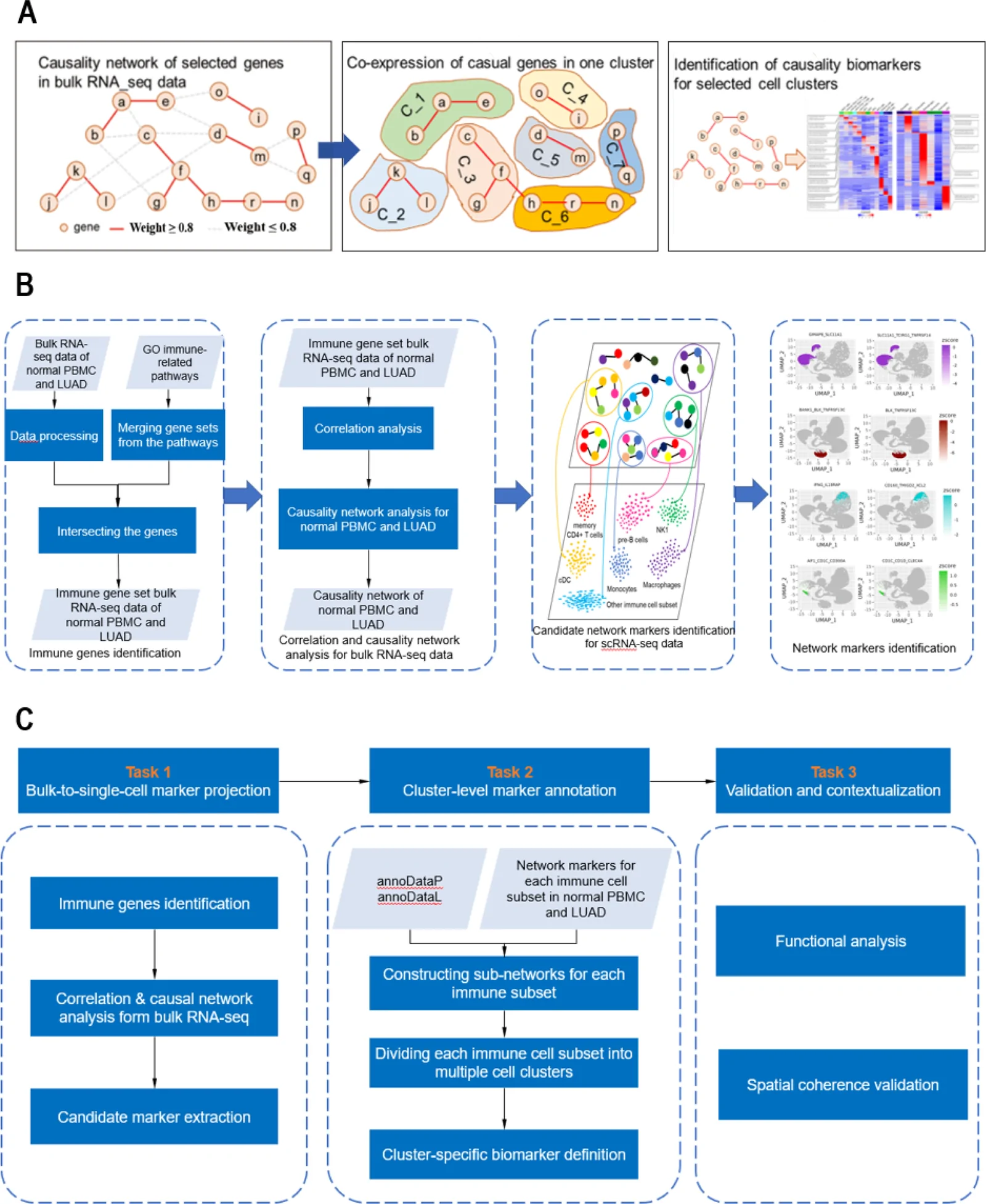
Overview of the network-informed analytical framework. **(A)** Hypothesis: Immune cell functional states can be defined by projecting bulk-derived network structures into single cell and spatial contexts. **(B)** Algorithmic workflow: Construction of Bayesian network-derived regulatory structures from bulk RNA-seq data, extraction of high confidence directed gene triplets as network biomarkers, and define structure-driven immune subclusters by projection network biomarkers onto scRNA-seq data. **(C)** Analysis process for three major tasks: (1) construction of gene level network structures and extraction of structure-based network biomarkers from bulk data; (2) definition of immune cell functional states by projecting biomarkers onto scRNA-seq data; and (3) assessment of functional relevance and spatial coherence of structure defined states using spatial transcriptomics.

To systematically characterize the network structure underlying immune cell functional states and their spatial organization within tissue architecture, we established a structure-driven framework for cell state identification and interaction inference. This framework is grounded in the following hypothesis: genes that exhibit stable network-derived directional associations at the bulk RNA-seq level are more likely to be co-expressed within the same cell, forming structurally coherent co-expression units. This hypothesis and its cross-scale implementation strategy are summarized in the overall framework shown in **Figure 1**. At single cell resolution, such regulatory modules can serve as network-informed features of cell identity, forming a network biomarker system for cell state definition. Furthermore, if two distinct cell clusters are characterized by network biomarkers linked through Bayesian network-derived associations, these clusters are more likely to engage in functional interaction or exhibit spatial proximity within the tissue context (**Figure 1A**). This model outlines a hierarchical reasoning path that spans from gene level network-derived associations to single cell state specification and tissue level interaction prediction.

To evaluate this hypothesis, we implemented a cross-scale modeling workflow spanning bulk, single cell, and spatial transcriptomics (**Figure 1B**). First, Bayesian network-derived regulatory structures were constructed from bulk RNA-seq data (Methods 4.3 Bayesian network structure analysis), and high-confidence directed subgraphs, which consists of 2 or 3 genes, were extracted as structural units (network markers). These network markers were then projected onto scRNA-seq expression matrices (Methods 4.5 Network marker identification for immune cell subsets) to identify co-expressed regulatory modules at the single cell level, and the stability of co-expression across clusters was used to define network biomarkers (Methods 4.6 Immune cell network biomarker system establishment). Finally, we mapped the corresponding cell clusters to spatial transcriptomic coordinates (Methods 4.10 Network biomarker relationship analysis) and assessed whether network-associated cell clusters showed spatial proximity.

This analytical strategy comprises three major tasks (**Figure 1C**): (1) construction of gene level network structures and extraction of structural units in bulk data; (2) identification of co-expressed network biomarkers and definition of functional states in single cell data; and (3) assessment of functional relevance and spatial coherence of structure defined immune states. Based on this framework, we performed systematic analyses across the three layers to evaluate its applicability and interpretability in distinct physiological contexts. The scRNA-seq and spatial transcriptomic datasets were generated from prospectively collected samples by our group at West China Hospital of Sichuan University, whereas context-matched public bulk RNA-seq cohorts were used to derive population-level immune gene network structures.

### 2.2 Construction of Bayesian network-derived regulatory structures and identification of cell subtypes in healthy peripheral blood

**Figure 2:**
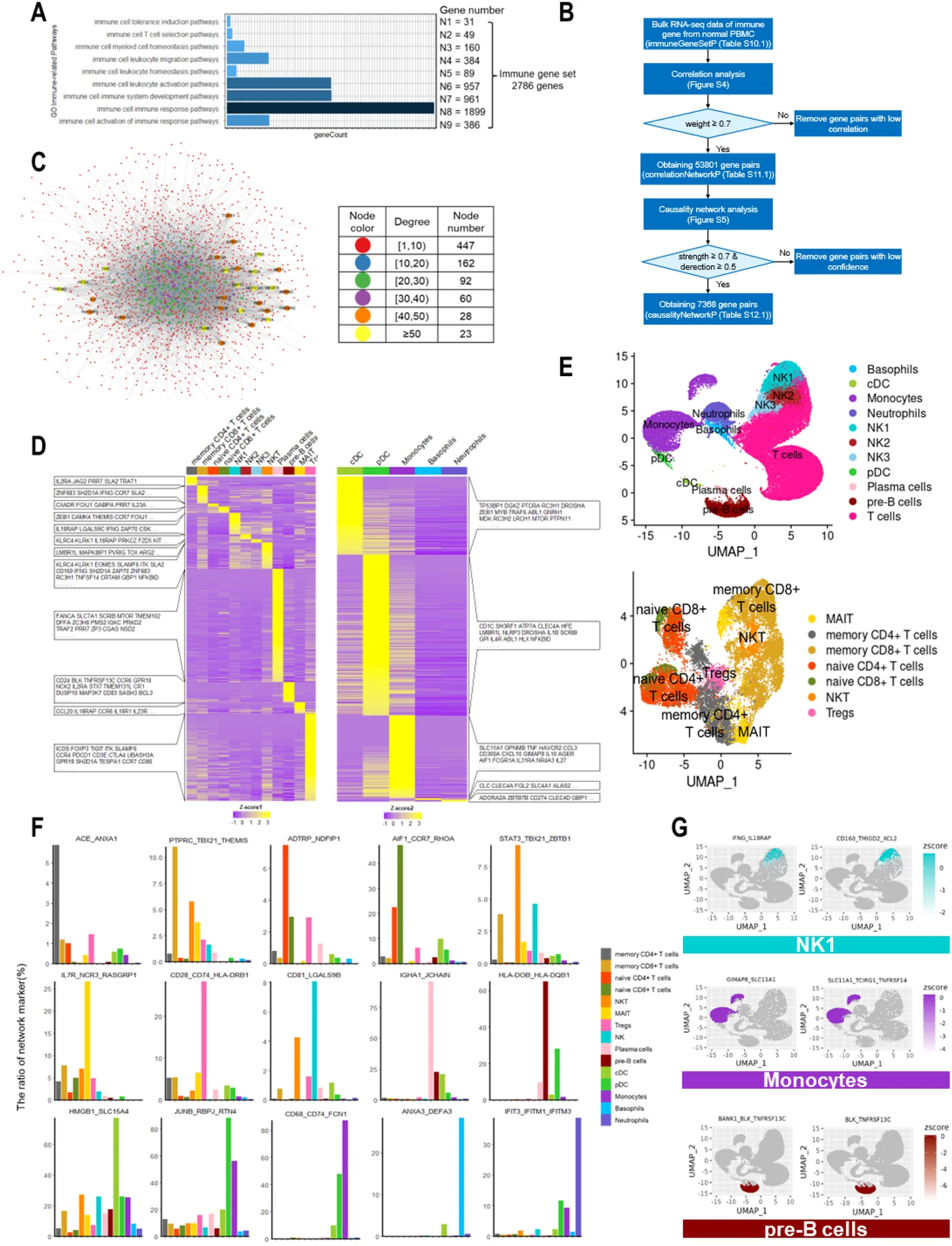
Bayesian network-derived structure construction and projection onto single cell data for PBMCs. **(A)** Selection of immune related genes from GO biological processes. **(B)** Correlation filtering and Bayesian network structure construction using bulk RNA-seq from normal PBMCs. **(C)** Visualization of the PBMC Bayesian network-derived structure, highlighting hub regulators and network topology. **(D)** Heatmap of binary co-expression of network markers across immune cell subsets. **(E)** UMAP visualization of PBMC immune cell subsets with representative network marker scores. **(F)** Usage score distribution of top-ranking network markers in PBMC subsets, indicating labeling specificity. **(G)** Distribution of network biomarker usage scores across cell types, illustrating subset specificity.

To decode the regulatory architecture underlying the immune system, we constructed Bayesian network-derived regulatory structures using bulk RNA-seq data from peripheral blood mononuclear cell (PBMC) populations derived from healthy donors (**Figure 2A**). A weighted correlation network was first generated via WGCNA, and modules significantly enriched in Gene Ontology (GO) immune related processes were retained. This resulted in a refined set of 2,553 immune genes for downstream network inference (**Supplementary Table S1.1**), encompassing key components involved in inflammation, antigen presentation, and lymphocyte signaling pathways.

Bayesian network modeling was performed by Hill-Climbing algorithm (34) for structure search and Bayesian Information Criterion (BIC) for scoring. To evaluate network robustness, we performed 200 bootstrap replicates and retained only edges if its confidence score is greater than or equal to 0.7. The final Bayesian network-derived structure comprised 7,368 high confidence gene–gene regulatory links (**Figure 2B**; **Supplementary Table 2.1**). The resulting network exhibited a non-random and modular topology, with central transcriptional regulators such as SPI1 and IRF4 acting as hubs across multiple pathways, reflecting biologically plausible coordination of immune processes.

From this global network, we extracted all directed subgraphs composed of 2 or 3 genes and defined them as candidate network markers. Each subgraph represents a minimal directed network motif, which is presumed to form a co-regulated module at the single cell level. A total of 2,816 such structures were identified and filtered by GO sub-pathway enrichment to yield a high confidence subset of immune relevant markers (**Figure 2C**).

We next projected these network markers onto a single cell RNA-seq dataset consisting of 36,753 PBMCs. For each marker, a binary co-expression score was computed for each cell, where full co-expression was defined as all constituent genes being expressed above TPM > 0.1. This yielded a binary cell by marker matrix capturing the structural expression landscape across the entire cell population (**Figure 2D**). Notably, many markers showed cluster restricted expression, suggesting that functional cell subtypes are constrained by discrete regulatory structures.

To define these states, we calculated the cluster specific usage rate of each marker—the fraction of cells in a given cluster exhibiting full marker co-expression. Markers with a usage rate ≥ 80% were designated as network biomarkers, providing structural signatures of cell identity. Across PBMCs, 19 immune cell subtypes were identified, each with a distinct panel of network biomarkers. These included highly specific modules such as CCL5–STAT1–IRF7, enriched in activated CD8^+^ T cells, and CD74–HLA-DRA–HLA-DPB1, broadly shared among antigen presenting cell subsets (**Figure 2E**; **Supplementary Figure S1A**).

A co-expression heatmap revealed clear structural separability between clusters (**Figure 2F**). A comprehensive analysis of network biomarker usage across all immune subtypes in PBMCs is provided in **Supplementary Figure S1A**, highlighting greater marker density in cDCs, naïve B cells, and regulatory T cells (**Supplementary Table S3.1**). Interestingly, a subset of markers was recurrently expressed across multiple related lineages, suggesting the presence of shared regulatory circuits underpinning immune functionality.

Unlike conventional marker gene–based methods, which define cell subtypes via differential expression of individual genes, our approach uses co-expressed network marker modules as structural units for immune cell state interpretation, thereby providing a complementary layer for annotating immune cell subtypes.

Taken together, these results demonstrate that bulk-derived Bayesian network structures can be systematically projected onto single cell resolution, where they define discrete and interpretable immune cell subtypes. These network biomarkers provide a structure-informed framework for immune cell state interpretation and for exploring network-level perturbations in disease contexts.

### 2.3 Evaluation of network-derived regulatory structure mapping in the tumor immune system

**Figure 3:**
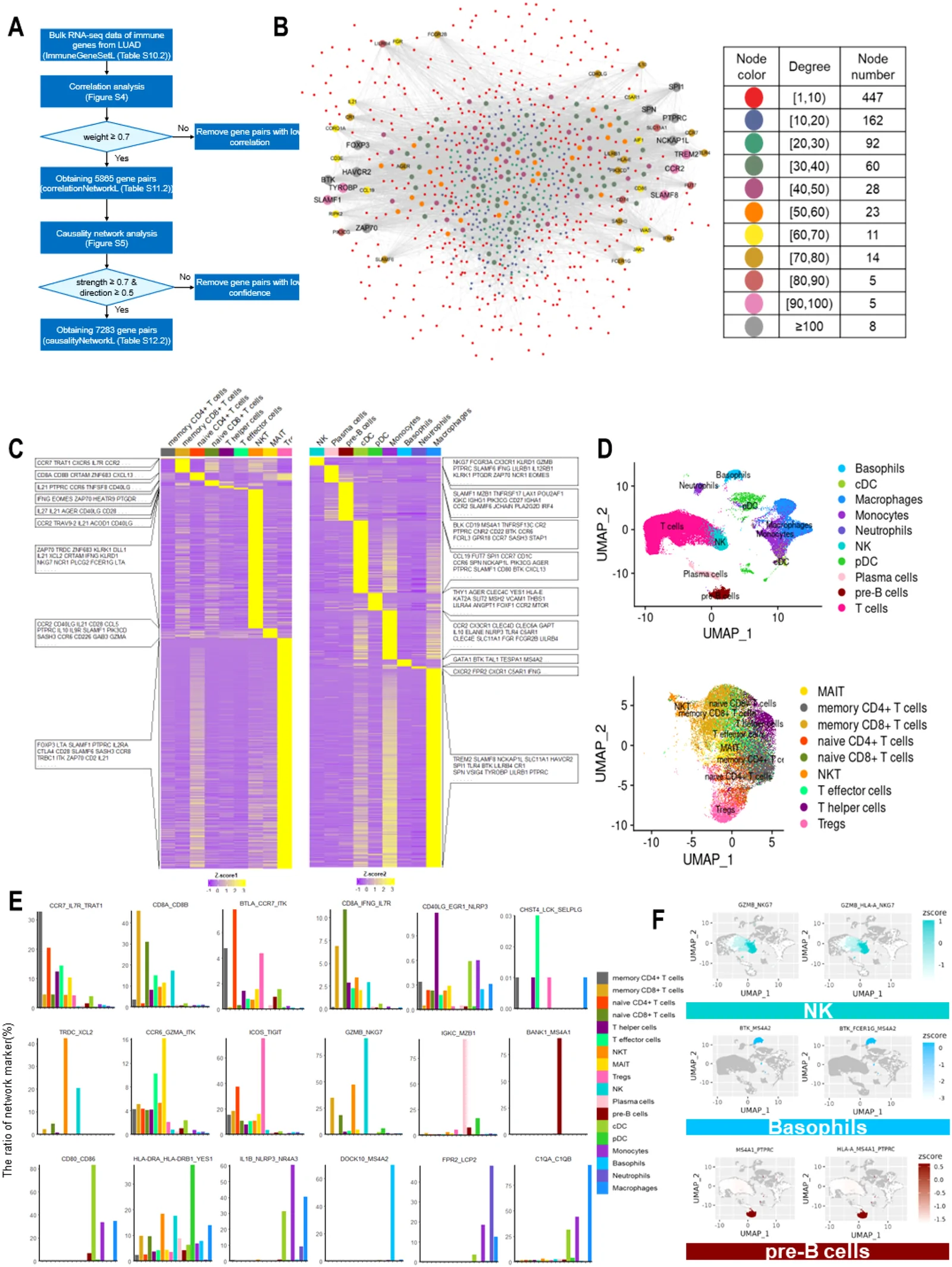
Bayesian network-derived regulatory structure reconstruction and marker projection in LUAD immune cells. **(A)** Correlation and Bayesian network inference for immune gene sets using LUAD bulk RNA-seq data. **(B)** Visualization of the LUAD Bayesian network-derived structure with node degree and hub connectivity. **(C)** Heatmap of network marker co-expression patterns across LUAD immune subsets. **(D)** UMAP visualization of LUAD immune cell subsets with representative network marker scores. **(E)** Usage score distribution of top-ranking network markers in LUAD subsets, indicating labeling specificity. **(F)** Distribution of network biomarker usage scores across cell types, illustrating subset specificity.

To evaluate the applicability of our framework under pathological conditions, we applied it to lung adenocarcinoma (LUAD) samples, reconstructing immune regulatory structures within the tumor microenvironment (**Figure 3A**). Bulk RNA-seq data from TCGA–LUAD were processed using the same immune gene set and WGCNA-GO enrichment pipeline, resulting in the selection of 873 immune related genes (**Supplementary Table S1.2**). Using the Hill-Climbing algorithm with BIC scoring and 200 bootstrap replicates, we constructed Bayesian network-derived regulatory structures comprising 7,283 high confidence regulatory edges (**Figure 3B**; **Supplementary Table S2.2**).

Compared to the PBMC derived network, the LUAD regulatory structure exhibited reduced edge density, and several key immune regulators, including SPI1 and IRF7, showed decreased node connectivity (**Figure 3C**). These features suggest localized disruptions in regulatory signaling within the tumor context. Nevertheless, the framework was able to recover network marker patterns in the LUAD context, supporting its applicability to tumor-derived data.

Network markers extracted from the LUAD Bayesian network-derived graph were projected onto a single cell RNA-seq dataset comprising 386,030 tumor infiltrating immune cells. Co-expression was assessed at the single cell level following the same criteria used in PBMC (**Figure 3D**). Using cluster specific usage thresholds, network biomarkers were identified across multiple immune subtypes. Although the overall co-expression frequency and marker abundance were reduced relative to PBMC, structure driven state definition remained feasible. Some clusters showed sparser marker coverage and less sharply defined boundaries (**Figure 3E**; **Supplementary Table S3.2**).

Further inspection revealed increased transcriptional noise and structural heterogeneity within several LUAD cell subsets, contributing to lower biomarker usage rates and reduced clustering stability (**Figure 3F**; **Supplementary Figure S1B**). These observations likely reflect the dynamic and perturbed immune architecture in tumor settings and indicate that network marker usage may vary across biological contexts.

Notably, despite such variability, the framework retained the ability to detect functionally relevant regulatory modules. For instance, the antigen presentation related marker CD74–HLA-DRA–HLA-DPB1 remained co-expressed within defined APC subsets, suggesting that some network marker patterns can still be observed in tumor immune contexts despite increased heterogeneity.

Taken together, these findings demonstrate that our approach is extensible to tumor derived data and capable of identifying interpretable regulatory units and immune cell subtypes at single cell resolution. This highlights its potential utility in modeling structure informed immune dynamics across normal and diseased tissues.

### 2.4 Structure-based identification of functional heterogeneity within immune cell subtypes

Building on our ability to resolve cell types across biological conditions, we next sought to determine whether the inferred regulatory structure could also delineate finer grained cell subtypes within defined immune lineages. While conventional single cell analysis primarily focuses on the identification of cell types, functional heterogeneity frequently arises within annotated immune subpopulations, especially in complex or pathological tissues. To address this, we extended our structure-driven framework to model cell subtypes within immune subtypes and evaluated its applicability across both normal and tumor contexts.

Focusing on conventional dendritic cells (cDCs), we first analyzed PBMC derived cells. For each cell, we constructed a binary matrix encoding whether each network biomarker was co-expressed (i.e., all constituent genes expressed above a TPM threshold). This structure usage matrix served as input for unsupervised clustering. The resulting clusters revealed distinct network biomarker usage patterns, indicative of multiple structure informed states within the cDC population (**Supplementary Figure S2A**).

To assess whether this structure defined clusters corresponded to functional differences, we performed differential gene expression (DEG) analysis followed by Gene Ontology enrichment (Methods 4.8 Differential gene expression and functional analysis) (**Supplementary Table S4.1**). The resulting clusters exhibited divergence in pathways related to antigen presentation, Toll-like receptor signaling, and chemokine mediated responses. Notably, this classification approach bypasses reliance on highly variable genes or predefined marker sets, instead leveraging network-derived regulatory structure logic to define functionally relevant states.

We then applied the same procedure to cDCs from LUAD samples. Despite increased transcriptional variability and network sparsity in the tumor microenvironment, we successfully identified structure informed clusters (**Supplementary Figure S2B**). Cluster-specific structure usage patterns were preserved, and both DEG and pathway enrichment analyses revealed distinct functional profiles (**Supplementary Table S4.2**), with several overlaps to PBMC derived clusters, suggesting biological consistency across contexts.

**Figure 4:**
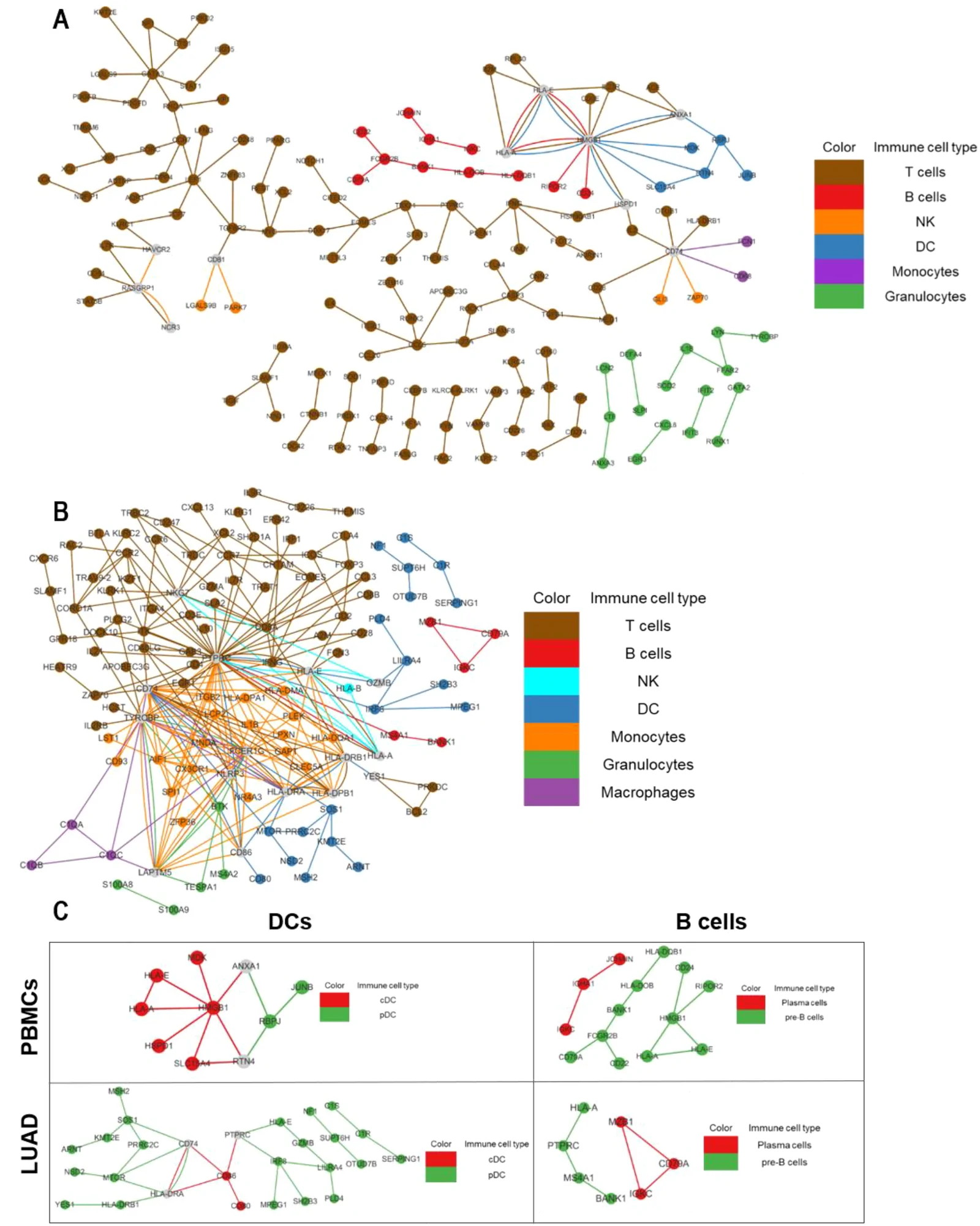
Identification of intra subtype functional states via network biomarkers. **(A)**Global visualization of the PBMC immune regulatory network and the node color indicates different immune cell types. **(B)** Network topology in LUAD, showing increased structural overlap and reduced subtype separation. **(C)** Representative examples of marker sharing subgraphs between PBMC and LUAD for DCs and B cells, illustrating tumor induced convergence of regulatory structure.

To evaluate the structural organization of immune regulatory programs under physiological and pathological conditions, we first examined the extent of network biomarker sharing among immune cell subtypes in PBMC. The pairwise sharing matrix revealed clearly defined inter subtype boundaries and minimal overlap of markers, indicating a high degree of regulatory specificity in the healthy immune system (**Figure 4A**). In contrast, LUAD derived immune cells exhibited broadly reduced marker usage across matched subtypes, suggesting widespread disruption of coordinated regulatory programs and structural complexity under tumor induced dysregulation (**Figure 4B**). To further illustrate the structural clarity observed in PBMC, **Figure 4C** shows a representative subregion between two closely associated subtypes, highlighting precise module usage without substantial redundancy.

In summary, our framework enables the resolution of intra lineage functional state heterogeneity by projecting network-derived regulatory structures onto the single cell space. This approach extends beyond cell type classification, offering a generalizable and interpretable strategy to uncover functional diversity within immune subtypes across diverse biological settings.

### 2.5 Functional heterogeneity of structure-driven immune cell subtypes revealed by transcriptomic profiling

To further assess the biological relevance of the structure-driven cell states identified in this framework, we combined differential expression with spatial distribution analyses across the identified states and cross-referenced each network-derived marker with its known immune function (**Supplementary Table S24**).

**Figure 5:**
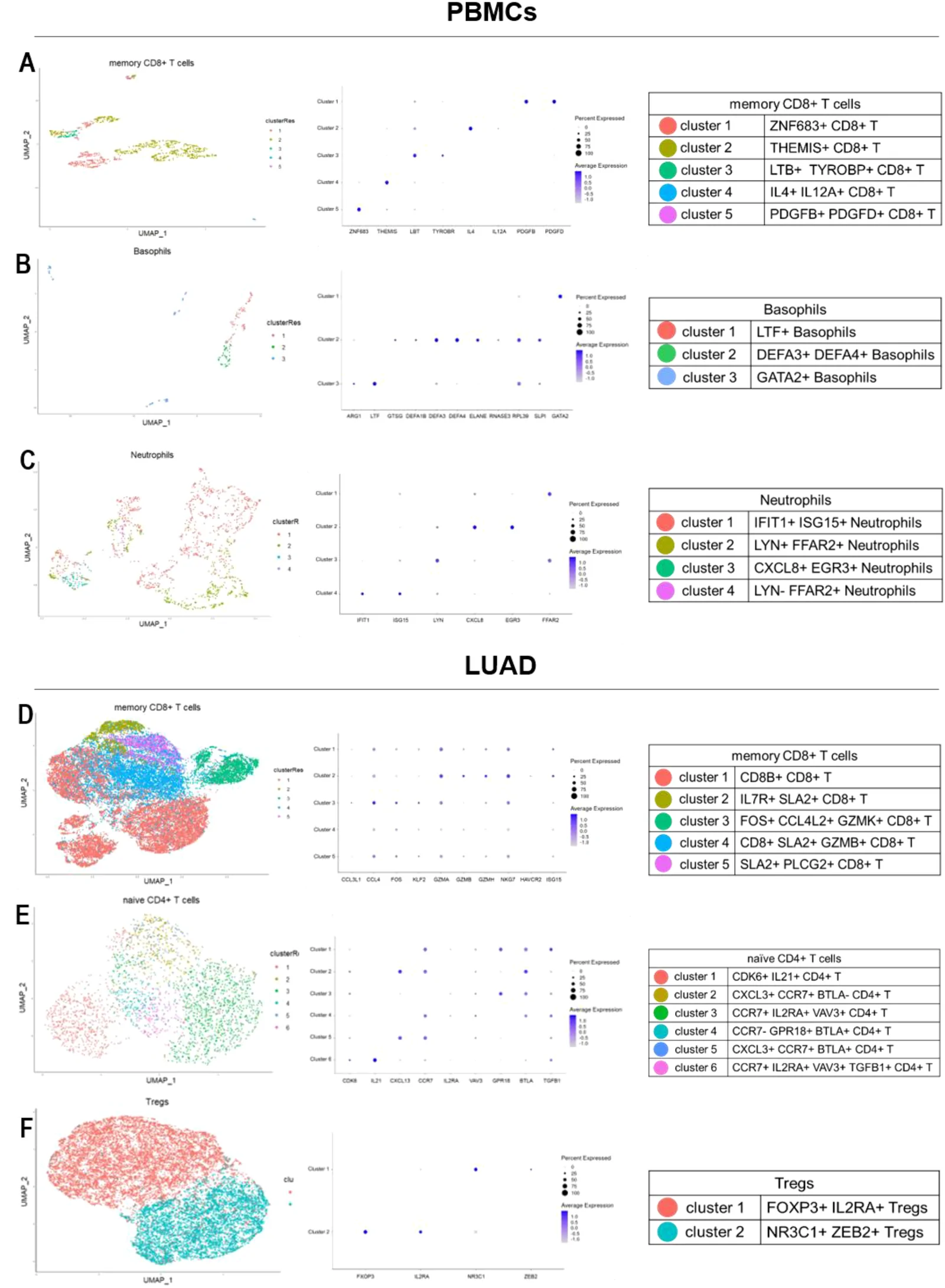
Structure-defined functional states across immune cell subtypes in PBMC and LUAD. UMAP projections, dot plots, and functional annotations of structure-defined clusters for memory CD8^+^ T cells **(A)**, basophils **(B)**, and neutrophils **(C)** in PBMC. Corresponding results for memory CD8^+^ T cells **(D)**, naive CD4^+^ T cells **(E)**, and Tregs **(F)** in LUAD. Alongside UMAPs and dot plots, a literature-backed table links each network-derived marker to established immune functions. Full citations and per-cluster summaries are provided in **Supplementary Table S24**.

Focusing first on immune cells from healthy peripheral blood mononuclear cells (PBMC), we selected three representative subsets: memory CD8+ T cells, basophils, and neutrophils, as examples. Using network biomarker-based stratification, we visualized the distribution of structure defined clusters within the UMAP embedding (**Figures 5A–C**). Distinct spatial segregation was observed among clusters, suggesting that regulatory structure can serve as an effective feature for functional state delineation.

We then characterized the molecular features of each cluster by performing differential gene expression (DEG) analysis and visualizing representative markers using dot plots (**Figures 5A–C**). In each plot, dot size represents the fraction of expressing cells within a cluster, and color intensity indicates average expression level. For example, in memory CD8+ T cells, Cluster 1 exhibited elevated expression of ZNF683 and GZMK, consistent with effector/tissue-resident memory programs, whereas Cluster 2 was enriched for THEMIS and LTB, aligning with altered TCR signaling thresholds and differentiation states. Similar functional divergence was observed among basophil and neutrophil clusters. As another example, neutrophil clusters split into an interferon-stimulated antiviral axis (e.g., IFIT1, ISG15) versus a signaling/chemotactic axis (e.g., LYN, FFAR2, CXCL8), recapitulating inflammatory versus homeostatic states. Based on these analyses, we summarized the network biomarker characteristics together with their literature-supported immune functions for each PBMC cluster (**Supplementary Table S24**), establishing a mechanistic framework for functional annotation of immune states under homeostatic conditions.

To examine the applicability of this approach in pathological contexts, we next applied it to immune cell populations from lung adenocarcinoma (LUAD) samples. Representative subsets, including memory CD8+ T cells, naive CD4+ T cells, Tregs, and basophils, were analyzed. Structure-driven clustering revealed clear segregation within the UMAP space even under tumor induced transcriptional variability (**Figures 5D–F**). Dot plot analyses further confirmed functional divergence among LUAD clusters (**Figures 5D–F**). For instance, memory CD8+ T cell Cluster 1 displayed high expression of CXCR4 and IFNG, indicative of an activated cytotoxic profile, whereas Cluster 2 was enriched for SELL and CCR7, suggesting a chemotactic and migratory function. Treg clusters were further defined by FOXP3/IL2RA-anchored suppressive programs with NR3C1 and ZEB2 providing functional specialization. Finally, we summarized the network biomarker profiles and their known immune functions for each LUAD cluster (**Supplementary Table S24**), highlighting the framework’s ability to resolve functional diversity across distinct immune lineages and tissue contexts. Together, these results demonstrate that structure-driven functional state identification enables a network-informed strategy for stratifying and literature-anchored annotation of functional heterogeneity across normal and pathological immune systems, offering a broadly applicable approach for high resolution immune ecosystem dissection.

### 2.6 Spatial coherence of network-defined immune cell subtypes in tissue architecture

To examine whether network-defined immune cell subtypes show spatial coherence in tissue sections, we analyzed spatial distances between cells labeled by network biomarker pairs in LUAD tissue sections.

We focused on two LUAD samples (C04140A5 and C04145D6) and categorized network biomarker pairs into two groups based on their Bayesian network-derived associations. Group 1 included biomarker pairs with strong Bayesian network-derived associations (bootstrap confidence ≥ 0.7), while Group 0 included those without such connections. For each pair of biomarker-labeled clusters, we calculated the Euclidean distance between their spatial centroids.

**Figure 6:**
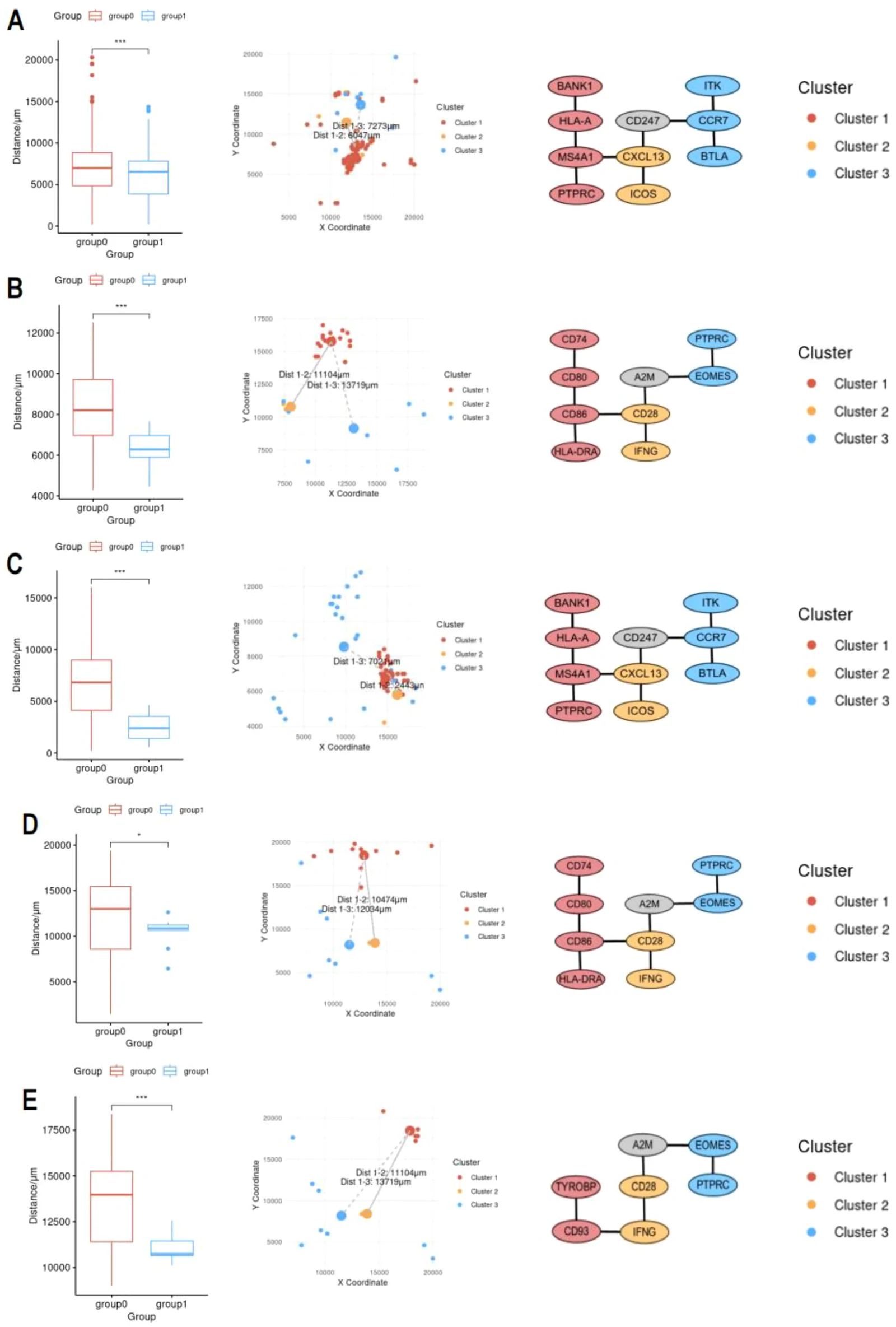
Spatial colocalization of network-defined immune clusters in LUAD. LUAD sample C04140A5: **(A)** Naïve CD4^+^ T cells and Pre-B cells; **(B)** Naïve CD8^+^ T cells and cDCs. LUAD sample C04145D6: **(C)** Naïve CD4^+^ T cells and Pre-B cells; **(D)** Naïve CD8^+^ T cells and cDCs; **(E)** Naïve CD8^+^ T cells and Monocytes. Each panel includes Left: Box plot compares the centroid distances between two cell clusters with (Group 1) or without (Group 0) strong Bayesian network-derived associations between their network biomarkers (confidence ≥ 0.7). Shorter distances in Group 1 suggest spatial colocalization associated with network-derived biomarker relationships; Middle: Spatial scatter plot shows the representative example of each cluster pair. Each dot represents a single cell. Lines connect the spatial centroids of two clusters: solid lines indicate strong network-derived associations (Group 1), and dashed lines represent no such association (Group 0); Right: Network biomarker composition of each cluster involved. Nodes represent biomarker genes, and edges represent Bayesian network-derived connections within each cluster. Grey nodes indicate genes shared across two clusters (cluster 2 and 3). A single symbol indicates P < 0.05, and three symbols indicate P < 0.001. The statistical comparison was performed using a t-test.

As shown in **Figures 6A, B** (sample C04140A5) and **Figures 6C–E** (sample C04145D6), cell clusters with network associations consistently showed statistically shorter centroid distances than unlinked clusters. These results suggest that bulk-derived network association is accompanied by spatial colocalization patterns within the tumor microenvironment.

To visually demonstrate this correspondence, we selected representative biomarker pairs from each group and mapped the spatial positions of the corresponding clusters. In the scatter plots (right panels), solid lines denote biomarker pairs with strong network-derived associations, while dashed lines indicate unconnected pairs. In all examples, centroids of strongly connected clusters were spatially closer, supporting the spatial coherence of network-defined cell states.

Despite the disorganized architecture and transcriptional noise characteristic of LUAD, this trend was observed consistently across both samples and all tested cell types (e.g., naïve CD4^+^ T cells, pre-B cells, cDCs, monocytes), highlighting the robustness of structure-informed spatial coherence.

Together, these findings show that bulk-derived network structures provide a cross-scale framework for linking immune gene co-expression, single-cell state definition, and tissue-level spatial organization in pathological contexts. Structure-defined cluster pairs connected by Bayesian network-derived associations exhibited shorter spatial distances than unlinked pairs, supporting spatial coherence between network-defined cell states.

## 3 Discussion

The primary objective of this study was to construct a network-informed analytical framework to comprehensively explore immune cell subtypes and functional heterogeneity within tumors and their microenvironment. Such complex biological systems, especially tumors, exhibit multilayered regulation and dynamic changes that cannot be fully captured by traditional single pathway and single molecule studies (35, 36).

Previous research typically focuses on individual molecules or pathways, which fail to integrate systemic regulatory information and often overlooks the complex interactions between environmental factors and cells (37, 38). This reductionist approach limits our ability to interpret cellular behavior in the context of tissue-level organization and disease dynamics. Thus, network-informed cross-scale analytical methods may help characterize higher-order regulatory structures and coordinated immune programs within the tumor microenvironment. To strengthen biological interpretability, we cross-referenced each network-derived marker with its established immune function (**Figure 5**; **Supplementary Table S24**). For instance, ZNF683/GZMK in memory CD8^+^ T cells correspond to effector/tissue-resident programs, while neutrophil markers IFIT1/ISG15 versus LYN/FFAR2/CXCL8 reflect interferon-stimulated versus chemotactic states. These examples illustrate that the recovered network structures align with well-established immune programs rather than data-driven artifacts.

Our approach integrates bulk RNA-seq, single-cell RNA-seq, and spatial transcriptomic data to establish a cross-scale network-informed framework for immune cell state interpretation. Rather than relying only on single-molecule markers, this framework uses coordinated network marker usage to provide an additional layer for annotating immune cell subpopulation states. The proposed strategy offers a structure-informed perspective for interpreting immune cell heterogeneity while remaining complementary to conventional expression-based approaches. Moreover, by incorporating spatial transcriptomic data, we evaluated whether network-defined immune states showed spatial coherence within tissue architecture, thereby providing additional insight into the spatial organization of the tumor immune microenvironment. The innovation of this study lies in linking bulk-derived network structure, single-cell state definition, and spatial organization within a unified analytical framework.

### 3.1 Advancements over previous methods

Existing single cell analysis methods, such as Seurat and other tools, typically rely on gene expression similarity to classify cell types and predict functional states (39). While these methods have made significant progress in cell type identification, their focus remains primarily on static gene expression patterns, without delving into the functional heterogeneity of cells and their regulatory mechanisms. For example, the Human Cell Atlas project classifies immune cells using surface markers, yet clustering methods based solely on expression features fail to reveal both the functional states of cells and the higher-order regulatory associations underlying coordinated immune cell programs (40). As a result, methods that integrate bulk RNA-seq and single cell RNA-seq data have made substantial progress in immunology and oncology, providing researchers with a deeper understanding of the functional heterogeneity of cells within the immune system and tumor microenvironment. For instance, the immune cell identification model proposed by Liu et al. (2020), which integrates single cell RNA sequencing with large scale gene expression data, effectively uncovers the composition and functional states of immune cell populations (41). This method has offered new approaches for the functional classification of immune cells and has made significant contributions to the identification and functional analysis of multiple immune cell subpopulations. Additionally, the method Scissor proposed by Sun et al. (2021) successfully combines bulk RNA-seq and single cell RNA-seq data to achieve fine grained immune cell subpopulation classification (42). By quantifying the similarity between each single cell and bulk sample, this method optimizes regression models to identify immune cell subpopulations associated with specific phenotypes. This approach has demonstrated significant potential in the functional classification of immune cell subpopulations in studies of lung cancer and melanoma, providing valuable insights for the clinical application of immunotherapy.

Existing immune cell state identification approaches can be broadly divided into several categories. First, unsupervised clustering workflows, represented by Seurat and related pipelines, identify cell populations based on transcriptomic similarity and then assign biological meaning through marker gene annotation (43, 44). Second, marker- or reference-based annotation methods, including atlas-guided strategies, classify cells by matching single-cell expression profiles to predefined cell type signatures or reference datasets (45, 46). Third, bulk–single-cell integration methods, such as Scissor, link single cells to bulk-level phenotypes by modeling the similarity between single-cell transcriptomes and bulk sample profiles (42). These approaches have greatly advanced immune cell annotation and phenotype association analysis, but they primarily use expression vectors, marker genes, or phenotype similarity as the main features for cell state definition.

However, despite offering initial insights into cellular heterogeneity within the immune system and tumor microenvironment, these studies predominantly rely on gene expression data to predict cell types and functional states and therefore provide limited information about higher-order gene dependency structures that may underlie coordinated immune cell programs. Although some methods integrate bulk and single-cell datasets, most of them use bulk information to identify phenotype-associated cells or infer cell type composition rather than to construct network-derived multi-gene markers for cell state definition (47).

Building on this, our study introduces a network-informed strategy that differs from existing approaches in three main aspects. First, the basic unit of state definition is not a single marker gene or an expression vector, but a network marker composed of two- or three-gene structures derived from bulk-level Bayesian network modeling. Second, these network markers are projected onto scRNA-seq data to define immune cell states through coordinated marker usage, thereby providing a structure-informed layer complementary to conventional expression-based clustering. Third, the resulting network-defined states are further examined using spatial transcriptomic data, allowing us to assess whether these states show spatial coherence within tissue architecture. Therefore, the proposed framework should be viewed not as a replacement for existing immune cell annotation methods, but as a complementary analytical mode that links bulk-level network structure, single-cell state definition, and spatial organization. Nevertheless, future systematic benchmarking against single-gene marker-based, module-score-based, and conventional expression-clustering approaches will be needed to quantitatively evaluate the discriminative performance and robustness of network-derived markers under different levels of single-cell sparsity and technical noise.

### 3.2 Limitations of the study

Despite the innovative and advantageous network-informed framework proposed in this study for the tumor immune microenvironment investigation, some limitations remain. First, although we combined bulk RNA-seq and single cell RNA-seq data, the stability of the regulatory network may be affected when the sample size is small or data heterogeneity is high. Although SCTransform normalization and Harmony-based batch correction were applied before clustering and network marker projection, residual technical biases related to sequencing depth, batch composition, and sample processing may still influence cell-state resolution and network marker usage, and should therefore be considered when interpreting context-specific differences. In addition, bulk-derived network structures are inherently context-dependent and may be influenced by tissue type, disease status, cellular composition, and the local microenvironment. Therefore, the PBMC- and LUAD-derived networks constructed in this study should be interpreted as context-specific, population-level network structures rather than universally transferable regulatory maps. To reduce direct context mismatch, PBMC- and LUAD-specific bulk networks were constructed separately and projected only onto single-cell datasets from the corresponding biological context. This is an inherent limitation of Bayesian network-based structure learning models, as they are prone to overfitting or bias when faced with highly variable data, particularly when data is sparse or noisy (47, 48). This study did not incorporate sparse learning methods such as LASSO or Ridge regression, mainly because the current dataset is sufficiently stable, and these methods typically require strong prior assumptions, which may constrain the model’s flexibility50. Nonetheless, future research could consider integrating these methods into the Bayesian network structure learning, especially when dealing with more complex or smaller datasets, to increase model stability.

Second, the learning process of Bayesian network structures involves high computational complexity, particularly when handling large gene sets and complex network structures, which may require substantial computational resources (49). Although this study did not involve extremely large-scale datasets, future research can optimize computational efficiency by improving learning algorithms. For instance, more efficient algorithms (such as random forests, gradient boosting machines, or deep learning models) can be employed to reduce computational complexity, or distributed computing techniques can be integrated to accelerate the processing of large datasets (50, 51). Modular modeling and multiscale modeling approaches could also further increase computational efficiency, enabling the model to handle more complex dataset.

Additionally, the current method of mapping single cell spatial transcriptomics data relies on binary co-expression determination. While providing some spatial information, it still has limitations in precision (52). In the future, continuous expression weighted models or deep learning methods (such as convolutional neural networks) could be incorporated to increase the accuracy of spatial localization, further improving the depiction of cellular spatial distribution and spatial relationships within the tumor immune microenvironment (53). A deeper analysis of spatial transcriptomics data could also reveal spatial organization between cells in the tumor immune microenvironment, providing new insights for targeted immune therapies.

Finally, the present study is primarily limited to PBMC and LUAD datasets. This scope reflects the current design of our pipeline: learning population-level network structures from context-matched bulk RNA-seq cohorts, projecting these structures onto single-cell data from the corresponding biological context, and evaluating spatial coherence using spatial transcriptomics. In this study, the bulk RNA-seq datasets used for Bayesian network construction were obtained from public cohort-level resources, whereas the scRNA-seq and spatial transcriptomic datasets were generated from samples collected by our team. Therefore, the current framework should be regarded as a context-matched, rather than donor-matched, cross-scale integration strategy. While public bulk RNA-seq cohorts provide useful population-level references for recurrent immune gene dependency patterns within a defined biological context, they cannot fully capture individual-level or cell-type-specific regulatory variation. We therefore prioritized a tightly controlled, prospectively collected cohort spanning a representative malignant immune microenvironment (LUAD) and a well-characterized non-malignant system (PBMC) to demonstrate cross-scale applicability. Consistent with a proof-of-concept objective, future work will expand to additional cancers and immune-related diseases as suitably donor-matched tri-modal datasets become available, enabling pan-cancer benchmarking, cross-cohort replication, and assessments of transferability to establish broader generalizability and clinical relevance. In addition, future studies incorporating longitudinal samples collected at multiple disease stages or treatment time points, together with larger and more diverse healthy control cohorts, will be important for evaluating the temporal stability, context dependence, and population-level robustness of the proposed framework.

### 3.3 Application prospects and future directions

The network-informed analytical framework proposed in this study offers an innovative analytical tool to investigate the tumor immune microenvironment and has broad interdisciplinary application potential. This framework not only provides insights into coordinated regulatory programs and spatial organization within the tumor immune microenvironment but also has the potential to be extended to the study of functional heterogeneity in other tissue systems, such as neural tissues, liver metabolic zones, and embryonic development systems. In these systems, the network-informed framework may help characterize relationships between cellular functional states and tissue structure under various physiological and pathological conditions, offering new perspectives for disease mechanism research. Furthermore, in tumor immunotherapy, the network-informed framework may support the identification of immune cell functional states, improve interpretation of tumor immune heterogeneity, and provide an analytical basis for exploring immunotherapy-related cellular programs. By deeply analyzing cell-cell interactions within the tumor microenvironment, this framework supports the development of more interpretable strategies for immune microenvironment analysis and potential therapeutic stratification.

In the future, the network-informed framework may be extended beyond tumor immune microenvironment research but also play a crucial role in other disease areas, particularly in neurodegenerative diseases, metabolic disorders, and cardiovascular diseases. Through interdisciplinary network-informed analysis, the framework is expected to help us identify key regulatory nodes in the progression of these diseases, characterize spatial organization between cells, thus promoting the development of precision medicine. As spatial transcriptomics data, single cell genomics, and large-scale genomic data continue to accumulate, the network-informed framework will be able to address more complex biological issues and provide robust data support for cross-tissue and cross disease research. Future integration with additional omics layers, such as single-cell ATAC-seq, proteomics, epigenomics, metabolomics, and ligand–receptor interaction analysis, may further improve the biological interpretation of network-defined cell states and help connect transcriptional programs with upstream regulatory mechanisms and downstream functional phenotypes. With the further enhancement of big data and computational power, cross-scale network-informed analysis may provide useful research methodologies in multiple research fields, offering new research methodologies and clinical application strategies for precision medicine and personalized treatments.

## 4 Methods

The scRNA-seq and spatial transcriptomic datasets analyzed in this study were generated from samples collected by the Institute of Thoracic Oncology, West China Hospital, Sichuan University. Bulk RNA-seq datasets used for Bayesian network construction were obtained from public cohort-level resources, including GEO GSE107011 for normal PBMCs and TCGA-LUAD for lung adenocarcinoma. These public bulk datasets were used to infer context-matched, population-level immune gene network structures, whereas the in-house scRNA-seq and spatial transcriptomic datasets were used for single-cell projection, immune cell state identification, and spatial coherence analysis. Detailed preprocessing procedures, QC metrics, and analytical parameters are provided in the Methods and **Supplementary Information**.

### 4.1 Single cell and bulk RNA-seq data processing

single cell RNA-seq data were obtained from the Institute of Thoracic Oncology at West China Hospital, Sichuan University, which consists of 36,753 normal peripheral blood mononuclear cells (PBMCs) from 4 samples (**Supplementary Table S5.1**) and 386,030 lung adenocarcinoma (LUAD) cells from 45 samples (**Supplementary Table 5.2**), respectively. Bulk RNA-seq data were obtained from public gene expression datasets, where the normal PBMC data (**Supplementary Table S6.1**) came from the Gene Expression Omnibus (GEO) (54) under accession code GSE107011 (55), and the LUAD data (**Supplementary Table S6.2**) came from TCGA (56). The process is shown in **Supplementary Figure S3**.

We preprocessed bulk RNA-seq data as follows: First, we removed samples whose column name suffix was not “_PBMC” in the normal PBMC dataset. Second, we removed low-expression genes, if their transcripts per million (TPM) values were less than 0.01. Third, we converted Ensembl IDs into gene symbols using the GENCODE GTF file (57) and used the calculated mean as the expression value for the same gene symbol within a sample. Fourth, we removed normal and non-frozen tissue samples in LUAD. Finally, we detected and removed outliers using the WGCNA R package (v1.71) (58). After preprocessing, we obtained 38,169 × 13 and 56,373 × 460 gene expression matrices from the normal PBMC (**Supplementary Table S7.1**) and LUAD (**Supplementary Table S7.2**) datasets, respectively.

### 4.2 Correlation analysis

We selected immune related pathways (**Supplementary Table S8**) from Gene Ontology (GO) database and merged the genes to construct an immune gene set for each pathway (**Supplementary Table S9**). After obtaining the immune gene sets, we used the corresponding bulk RNA-seq data of normal PBMC (**Supplementary Table 10.1**) and LUAD (**Supplementary Table S10.2**) to identify highly correlated immune genes. The process is shown in **Supplementary Figure S4**.

Firstly, we constructed gene correlation networks using the “adjacency” function of the WGCNA R package (v1.71) by setting the parameter to “power = 1”. Second, we filtered out low-correlation genes (Equation 1) if their Pearson correlation coefficient is less than 0.7 (59). Finally, we identified 2,553 and 873 high-correlation immune genes for the normal PBMC (**Supplementary Table S11.1**) and LUAD (**Supplementary Table S11.2**) datasets, respectively, which were used for further Bayesian network structure learning.

(1) $$\rho_{\left(X,Y\right)}=cov(X,Y)/(\sigma_{X}\sigma_{Y})$$

Here, X and Y are the gene expression vector of gene X and Y, respectively; $\rho_{X,Y}$ is the Pearson correlation coefficient between gene X and Y; σ is the standard deviation of gene expression vector of gene X or Y.

### 4.3 Bayesian network structure analysis

We built up gene regulatory network structures by Bayesian network structure learning (60), and calculated the confidence of the gene regulatory networks using the bootstrap algorithm (61–65). These procedures were implemented using the bnlearn R package (v4.8-20220314) (66). The process is shown in **Supplementary Figure S5**.

Firstly, we used the previously identified high-correlation immune genes from the bulk RNA-seq data of normal PBMC (**Supplementary Table S11.1**) and LUAD (**Supplementary Table S11.2**) as input. Secondly, we grouped the immune genes using GO enrichment analysis (67). For each GO-enriched gene set, Bayesian network structure learning was performed using the Hill Climbing algorithm as the network structure search strategy, and the Bayesian Information Criterion (BIC) was used as the scoring metric to select the optimal network structure (68, 69). In this procedure, model training refers to the iterative search of network structures that maximize the BIC score for each immune gene set.

To evaluate the stability of inferred edges, we performed 200 bootstrap replicates. In each replicate, Bayesian network structure learning was repeated, and the confidence of each edge was calculated according to its frequency of occurrence across bootstrap-derived networks. Following the confidence filtering strategy reported by Wolen et al., edges with confidence scores greater than or equal to 0.7 were retained as high-confidence edges (59). This bootstrap-based filtering step was used as the validation procedure to reduce unstable or weakly supported edges. Ultimately, we obtained Bayesian network-derived gene regulatory structures containing 7,368 gene pairs for normal PBMC (**Supplementary Table S12.1**) and 7,283 gene pairs for LUAD (**Supplementary Table S12.2**), which were used for downstream analyses.

### 4.4 Unsupervised clustering and immune cell type annotation

We performed data preprocessing, unsupervised clustering, and cell type annotation for the scRNA-seq data of normal PBMC (**Supplementary Table S5.1**) and LUAD(**Supplementary Table S5.2**) by Seurat R package (v4.2.0) (43). The process is shown in **Supplementary Figure S6**.

Firstly, we normalized the data using the “SCTransform” function with default parameters. SCTransform was applied to reduce technical variation related to sequencing depth and library size before downstream dimensionality reduction and clustering. Secondly, we performed principal component analysis (PCA) and selected the top 50 principal components (PCs) for clustering. Batch effects among samples were corrected using the Harmony algorithm before neighborhood graph construction. We then performed unsupervised clustering with the “FindNeighbors” and “FindClusters” functions, setting the resolution parameter to 0.8. For cell type annotation, we collected marker genes from the CellMarker database (70) and previous literature (41, 71). We used the “FeaturePlot” and “DotPlot” functions to visualize marker gene expression and manually annotated the clusters. In total, we annotated 15 and 18 immune cell types for normal PBMC (**Supplementary Table S13.1**) and LUAD (**Supplementary Table S13.2**), respectively.

After cell type annotation, the TPM-normalized expression matrix was further binarized for the projection of network biomarkers onto single-cell transcriptomic data. Genes with TPM > 0.1 were considered detectably expressed and were assigned a binary value of 1; otherwise, they were assigned a value of 0. This threshold was used as a permissive low-expression cutoff to generate binary inputs for network marker co-expression scoring, rather than as a strict criterion for defining biological expression or non-expression of individual genes. A cutoff around 0.1 TPM has been used in previous transcriptomic studies and large-scale resources as a low-expression or detectable-expression threshold (72–74). Because downstream analyses were performed at the multi-gene network-marker and cluster-proportion levels, the influence of stochastic dropout or low-level background expression of individual genes was expected to be mitigated by network-level aggregation.

### 4.5 Network marker identification for immune cell subsets

Before network marker identification, scRNA-seq data were first subjected to preprocessing, normalization, batch correction, and immune cell type annotation as described above. Network marker projection was therefore performed on preprocessed and annotated single-cell datasets rather than on raw or uncorrected cell clusters. In this study, a “network marker” was defined as a gene subnetwork derived from the Bayesian network and used to identify and distinguish immune cell subsets, whereas “co-expression” referred to the simultaneous expression of all nodes within a given gene network in an individual cell.

We constructed gene subnetworks by permuting the genes from the Bayesian network-derived gene regulatory structure with respect to its network topology, and the number of nodes for each sub-network is limited to 2 or 3. And then, we obtained network markers for each immune cell subset by calculating the scores of each sub-network. The process is shown in **Supplementary Figure S7**.

We applied the Bayesian network-derived gene regulatory structure and the annotated single cell RNA-seq data of normal PBMC (**Supplementary Tables S12.1**, **S13.1**) and LUAD (**Supplementary Tables S12.2**, **S13.2**) as input, respectively. Firstly, we divided the Bayesian network-derived structure into multiple subnetworks and calculated the ratio for each subnetwork in each cell subset (Equation 2) of normal PBMC (**Supplementary Table S14.1**) and LUAD (**Supplementary Table S14.2**), respectively. We then filtered and classified subnetworks: after obtaining subnetwork scores across all cell subsets, we applied Equation 3to select network markers and assign them to the subset with the highest score. Finally, we identified the network markers for each immune cell subset of normal PBMC (**Supplementary Table 15.1**) and LUAD (**Supplementary Table 15.2**), respectively.

(2) $$Ratio_{i,j}=\frac{n_{i,j}}{N_{j}}$$

(3) $$holdRatio=\bar{R}+3\sigma_{R}$$

Here, $n_{i,j}$ represents the number of cells which co-express sub-network i in immune cell subset j; $N_{j}$ represents the number of cells in immune cell subset j. $Ratio_{i,j}$ represents the score of sub-networks i in immune cell subset j. Here, $\bar{\text{R}}$ is the mean of ratios of immune cell subsets; ${\text{σ}}_{R}$ is the standard deviation of ratios of immune cell subsets. If the highest ratio of the sub-network is more than holdRatio, we set the sub-network as a network marker.

### 4.6 Immune cell network biomarker system establishment

After manually classifying single cell data into immune cell subsets, we compiled network marker sets for each subset. These sets were used to further divide immune cell subsets into clusters and establish a network biomarker system. The process is shown in **Supplementary Figure S8**.

We applied the annotated scRNA-seq data and network marker sets of normal PBMC (**Supplementary Tables S13.1**, **S15.1**) and LUAD (**Supplementary Tables S13.2**, **S15.2**) as input, respectively.

For normal PBMC, firstly, we defined the subset-specific network marker set as G and constructed a matrix(cellNetworkM), where rows represent cells and columns represent markers in G. Secondly, we determined whether each cell co-expressed the markers in G. We assign 1 to cellNetworkM if all genes were expressed, and 0 otherwise. Thirdly, we used cellNetworkM as input to a clustering algorithm (Algorithm S1) to divide the subset into multiple clusters. Fourthly, we computed the clusterRatio (Equation 2) for each network marker in each cluster. Fifthly, we selected network markers if their clusterRatio is greater or equal to 80%, and merged them to form a network biomarker if its overall clusterRatio is greater than or equal to 80%. Finally, we obtained the cluster assignment and the network biomarker for each cluster. The same procedure was applied to other immune cell subsets in the PBMC dataset to establish the network biomarker system (**Supplementary Table S16.1**).

For LUAD, due to variability introduced by tumor microenvironment, immune evasion, and tumor heterogeneity, additional procedures were applied. Firstly, we repeated the above steps to define G, construct cellNetworkM, apply Algorithm S1 to divide the immune cell subset into multiple cell clusters (orgClusterResL), as well as compute clusterRatio and obtain candidate biomarkers (orgCanBiomarkerL). We then used orgClusterResL and orgCanBiomarkerL as the input for Algorithm S2 to generate new clusters (midClusterResL), and repeated the marker selection procedure (midCanBiomarkerL) until midClusterResL matched orgClusterResL. We identified midClusterResL and midCanBiomarkerL as the final clustering results and network biomarker for the cell subset, respectively. Finally, we obtained the cluster assignment and the network biomarker for each cluster. The procedure was applied to all LUAD subsets to establish the network biomarker system (**Supplementary Table S16.2**).

### 4.7 Cell cluster visualization for immune cell subsets

To assess the distribution and biological significance of clusters within each immune cell subset, we visualized them by UMAP plots generated with the monocle3 R package (v1.3.1) (75). The process is shown in **Supplementary Figure S9**.

We applied the annotated scRNA-seq data and network biomarker system of normal PBMC (**Supplementary Table S13.1**, **S16.1**) and LUAD (**Supplementary Tables S13.2**, **S16.2**) as input, respectively. Firstly, we constructed the single cell RNA-seq counts matrix restricted to genes from network biomarkers for each immune cell subset. Next, we created a CellDataSet object by “new_cell_data_set” function, preprocessed the data by “preprocess_cds” function, and applied dimensionality reduction via “reduce_dimension” function by setting parameter reduction_method = ‘UMAP’. Two-dimensional coordinates were extracted and visualized by “plot_cells” function. The same procedure was applied to visualize clusters in each immune cell subset of normal PBMC (**Supplementary Table 17.1**) and LUAD (**Supplementary Table S17.2**), respectively.

### 4.8 Differential gene expression and functional analysis

To evaluate the functional relevance of network biomarkers, we performed differential gene expression (DGE) analysis and functional enrichment for each cluster within immune cell subsets. The process is shown in **Supplementary Figure S10**.

We applied the annotated scRNA-seq data and network biomarker system of normal PBMC (**Supplementary Tables 13.1**, **S16.1**) and LUAD (**Supplementary Tables S13.2**, **S16.2**) as input, respectively. The single cell RNA-seq counts matrix was converted into a Seurat object, followed by quality control and dimensionality reduction. We performed DGE analysis by Seurat’s “FindAllMarkers” function (v4.2.0) (43) and applied filters by setting adjusted p < 0.05 and log2(fold change) > 0.25. Enrichment analysis was performed using “enrichGO” from the clusterProfiler package (v4.4.4) (67) by setting parameters pvalueCutoff = 0.05, ont = ‘BP’, and OrgDb = org.Hs.eg.db. Clusters were defined based on the expression of representative DEGs from enriched pathways. The same process was applied to all immune cell subsets from PBMC (**Supplementary Table S18.1**) and LUAD (**Supplementary Table S18.2**), respectively.

### 4.9 Raw stereo-seq data processing

Stereo-seq raw data (**Supplementary Table S19**) were processed according to standard protocols (76). The process is shown in **Supplementary Figure S11**. CID sequences were mapped onto the coordinates of the *in-situ* capture chip, allowing for one base mismatch to correct sequencing or PCR errors. Reads with MID containing either N bases or >2 bases with a quality score <10 were removed. cDNA sequences with valid CID and MID were aligned to the GRCh38 human genome by STAR (77). Expression profiles were generated by CID information and aggregated from bin size 1 to 200, with coordinates assigned accordingly (**Supplementary Table S20**).

### 4.10 Unsupervised clustering and cell type annotation for stereo-seq data

After obtaining the expression matrix (**Supplementary Table S20**), normalization, scaling, and clustering were performed by Seurat (v4.2.0) (78). Marker genes were retrieved from CellMarker (70) and previous literature (41, 71). Cluster annotation was performed by visualizing the expression of marker genes. Immune clusters were further subdivided and annotated to identify more refined immune cell types (**Supplementary Table S21**). The process is shown in **Supplementary Figure S12**.

### 4.11 Network biomarker relationship definition

After establishing the network biomarker system of LUAD (**Supplementary Table S16.2**), we defined relationships between biomarkers from different immune subsets as follows:

If a gene pair with strong Bayesian network-derived association existed between two network biomarkers and the gene pair was not co-expressed in either cluster, a strong inter-biomarker network relationship was defined. Otherwise, the biomarkers were considered independent. Network-derived relationships were derived from the established gene regulatory network (**Supplementary Table S22**). The process is shown in **Supplementary Figure S13**.

### 4.12 Network biomarker relationship analysis

After annotating immune cells (**Supplementary Table S21**), we identified those mapped by network biomarkers (**Supplementary Table S16.2**) for each subset. For biomarker pairs across subsets, we calculated the average cell-to-cell distance. Pairs were grouped into those with and without strong Bayesian network-derived associations. A T-test (16, 52, 63, 79–83) was then performed to assess whether biomarker pairs with strong network-derived associations exhibited statistically shorter cell-to-cell distances than those without such a relationship (**Supplementary Table S23**). The process is shown in **Supplementary Figure S14**.

## Data Availability

The datasets presented in this study can be found in online repositories. The names of the repository/repositories and accession number(s) can be found in the article/**Supplementary Material**.
