# Supplementary material for: Network-informed deconvolution of bulk immune gene co-expression reveals single-cell programs and spatial organization
Source: Front Immunol. 2026 Sep 1;17:1843602. doi: 10.3389/fimmu.2026.1843602 (PMC13574685; doi:10.3389/fimmu.2026.1843602)
Supplement: Supplementary file 1 [file Table1.docx]

**SUPPLEMENTAL INFORMATION FOR**

**Network-Informed Deconvolution of Bulk Immune Gene Co-expression Reveals Single-cell Programs and Spatial Organization**

content

[1. Supplementary Figures	3](#_Toc16856)

[1.1 Marker gene expression profiles	3](#_Toc24699)

[1.2 Expanded view of structure usage and marker sharing	4](#_Toc6915)

[1.3 Single-cell and bulk RNA-seq data processing	5](#_Toc5257)

[1.4 Correlation analysis	6](#_Toc10056)

[1.5 Causality network analysis	7](#_Toc16643)

[1.6 Unsupervised clustering and immune cell type annotation	8](#_Toc20000)

[1.7 Network markers identification for immune cell subsets	9](#_Toc26449)

[1.8 Immune cell network biomarker system establishment	10](#_Toc11220)

[1.9 Cell clusters visualization for immune cell subsets	11](#_Toc30967)

[1.10 Differential expressed genes identification and functional analysis	12](#_Toc8373)

[1.11 Raw Stereo-seq data processing	13](#_Toc7800)

[1.12 Unsupervised clustering and immune cell type annotation of Stereo-seq data	14](#_Toc7908)

[1.13 Network biomarker relationship defining	15](#_Toc26227)

[1.14 Network biomarker relationship analysis	16](#_Toc21376)

[2. Supplementary Algorithms	17](#_Toc9881)

[2.1 Immune cell subset division method based on kmeans algorithm	17](#_Toc28224)

[2.2 Immune cell subsets of LUAD division method based on Algorithm S1	18](#_Toc25964)

[3. Supplementary Tables	19](#_Toc8883)

[3.1 Correlation matrices used for immune gene filtering	19](#_Toc6892)

[3.2 Bayesian gene-gene regulatory pairs	19](#_Toc6343)

[3.3 Network marker usage ratios across cell subsets	19](#_Toc18323)

[3.4 Summary statistics for immune cell subset biomarker systems	20](#_Toc28230)

[3.5 Raw single-cell RNA-seq data profiles	20](#_Toc7252)

[3.6 Raw bulk data profiles	20](#_Toc14691)

[3.7 Gene expression matrices from the normal PBMC and LUAD datasets	21](#_Toc892)

[3.8 Immune-related pathway curation	21](#_Toc15505)

[3.9 Immune gene set definition	21](#_Toc6560)

[3.10 Immune gene set bulk RNA-seq data	21](#_Toc15932)

[3.11 results of Correlation analysis	22](#_Toc24320)

[3.12 results of Causality network analysis	22](#_Toc19226)

[3.13 Unsupervised clustering and immune cell type annotation	22](#_Toc27914)

[3.14 Candidate network marker identification	22](#_Toc8236)

[3.15 Candidate network marker identification	23](#_Toc6368)

[3.16 Cell clustering results across immune subsets	23](#_Toc8620)

[3.17 Cell cluster visualization	23](#_Toc29444)

[3.18 Functional definition of immune subclusters	24](#_Toc11892)

[3.19 Raw Spatial transcriptome data from the Stereo-seq platform	24](#_Toc21930)

[3.20 Processed spatial transcriptome data from the stereo-seq platform	24](#_Toc23465)

[3.21 Unsupervised clustering and annotation of spatial transcriptome data	24](#_Toc32309)

[3.22 Causal relationships between network biomarkers	25](#_Toc17069)

[3.23 Spatial analysis of causally linked biomarker-defined clusters	25](#_Toc21473)

[3.24 Marker genes information	25](#_Toc7072)

[4. Supplementary Codes	26](#_Toc19646)

1. **Supplementary Figures**

**1.1 Marker gene expression profiles**

**Figure S1.** Marker gene expression profiles supporting immune cell subset annotation. (A–B) UMAP projections overlaid with representative marker gene expression patterns for each annotated immune cell subset in PBMC (A, from Figure 2) and LUAD (B, from Figure 3). This figure complements the cell type annotations shown in the left panels of Figure 2 and Figure 3, with full marker expression maps displayed separately for clarity.

**1.2 Expanded view of structure usage and marker sharing**

**Figure S2.** Expanded view of structure usage and marker sharing across immune cell subsets. (A–B) Stacked bar plots showing the distribution of network biomarker usage among structure-defined clusters within each annotated immune cell subset in PBMC (A) and LUAD (B).

**1.3 Single-cell and bulk RNA-seq data processing**

We illustrate how to process bulk RNA-seq data in the main manuscript.

**Figure S3.** The procedure to process bulk RNA-seq data.

**1.4 Correlation analysis**

We illustrate how to carry out correlation analysis in the main manuscript.

**Figure S4.** The procedure to carry out correlation analysis.

**1.5 Bayesian network structure analysis**

We illustrate how to perform Bayesian network structure analysis in the main manuscript.

**Figure S5.** The procedure to carry out Bayesian network structure analysis.

**1.6 Unsupervised clustering and immune cell type annotation**

We illustrate how to carry out unsupervised clustering and immune cell type annotation in the main manuscript.

**Figure S6.** The procedure to carry out unsupervised clustering and immune cell type annotation.

**1.7 Network markers identification for immune cell subsets**

We illustrate how to identify network markers for immune cell subsets in the main manuscript.

**Figure S7.** The procedure to identify network markers for immune cell subsets.

**1.8 Immune cell network biomarker system establishment**

We illustrate how to establish immune cell network biomarker system in the main manuscript.

**Figure S8.** The procedure to establish immune cell network biomarker system.

**1.9 Cell clusters visualization for immune cell subsets**

We illustrate how to visual cell clusters for immune cell subsets in the main manuscript.

**Figure S9.** The procedure to visual cell clusters for immune cell subsets.

**1.10 Differential expressed genes identification and functional analysis**

We illustrate how to identify differential expressed genes and carry out functional analysis in the main manuscript.

**Figure S10.** The procedure to identify differential expressed genes and carry out functional analysis.

**1.11 Raw Stereo-seq data processing**

We illustrate how to process raw stereo-seq data in the main manuscript.

**Figure S11.** The procedure to process raw stereo-seq data

**1.12 Unsupervised clustering and immune cell type annotation of Stereo-seq data**

We illustrate how to carry out unsupervised clustering and immune cell type annotation of Stereo-seq data in the main manuscript.

**Figure S12.** The procedure to carry out unsupervised clustering and immune cell type annotation of Stereo-seq data.

**1.13 Network biomarker relationship defining**

We illustrate how to define network biomarker relationship in the main manuscript.

**Figure S13.** The procedure to define network biomarker relationship.

**1.14 Network biomarker relationship analysis**

We illustrate how to analysis network biomarker relationship in the main manuscript.

**Figure S14.** The procedure to analysis network biomarker relationship.

2. **Supplementary Algorithms**

**2.1 Immune cell subset division method based on kmeans algorithm**

**Algorithm S1****:** Immune cell subset division method based on kmeans algorithm

Input: cellNetworkM, The lowest proportion of cells in clustering results (cellRatioLow), The lowest clusterRatio in high expression network marker (highNetworkMarkerRatioLow)

Output: Clustering results (clusterRes)

Procedure:

1. Preprocessing cellNetworkM;
2. Set cellRatioLow = 80%;
3. Set highNetworkMarkerRatioLow = 80%;
4. Calculating the number of cells in cellNetworkM (allCellCount);
5. Initializing the clustering results by defining cellNetworkM as a cluster, orgClusterRes = {cluster1 };
6. while TRUE: # Looping reading clusters in orgClusterRes
7. Obtaining the first cluster in orgClusterRes (cluster1);
8. if the number of cells in cluster1 <= allCellCount*0.01:
9. break;
10. end if:
11. Calculating the clusterRatio for each network marker in cluster1 (Equation 2);
12. Counting the number of network markers in cluster1 with clusterRatio>=highNetworkMarkerRatioLow (highNetworkMarkerCount);
13. if highNetworkMarkerCount > 0:
14. Appending cluster1 to the final clustering results (clusterRes);
15. Removing cluster1 from orgClusterRes;
16. Sorting clusters in orgClusterRes in descending order based on the number of cells;
17. Calculating the number of cells in cluster1 (cellCount);
18. if cellCount/allCellCount >= cellCountRatioLowest:
19. Break;
20. else:
21. Employing kmeans algorithm to divide cluster1 into two clusters (cluster1A and cluster1B);
22. Removing cluster1 from orgClusterRes;
23. Appending cluster1A and cluster1B to orgClusterRes;
24. Sorting clusters in orgClusterRes in descending order based on the number of cells;
25. end if;
26. end while;
27. Obtaining the final clustering results (clusterRes).

**2.2 Immune cell subsets of LUAD division method based on Algorithm S1**

**Algorithm S2:** Immune cell subsets of LUAD division method based on Algorithm S1

Input: orgClusterResL, orgCanBiomarkerL, The lowest clusterRatio in high expression network marker (highNetworkMarkerRatioLow), The highest clusterRatio in low expression network marker (lowNetworkMarkerRatioHigh)

Output: Clustering results (clusterResL)

Procedure:

1. Set highNetworkMarkerRatioLow = 80%;
2. Set lowNetworkMarkerRatioHigh = 50%;
3. Calculating the number of cells in orgClusterResL (allCellCount);
4. Sorting clusters in orgClusterResL in descending order based on the number of cells, orgClusterResL = {cluster1, cluster2, ……, clustern};
5. Calculating the clusterRatio for each candidate network biomarker from orgCanBiomarkerL in each cluster (Equation S4);
6. while TRUE: # Looping reading clusters in orgClusterResL
7. Obtaining the first cluster in orgClusterResL (cluster1);
8. Obtaining candidate network biomarkers from orgCanBiomarkerL in cluster1 with lowNetworkMarkerRatioHigh <= clusterRatio <= highNetworkMarkerRatioLow (specialCanBiomarker);
9. if specialCanBiomarker != {}:
10. Merging candidate network biomarkers from specialCanBiomarker (mergeCanBiomarker)
11. Dividing cluster1 into two clusters based on whether cell co-expresses mergeCanBiomarker or not (cluster1A and cluster1B);
12. Removing cluster1 from orgClusterResL;
13. Appending cluster1A and cluster1B to clusterResL;
14. else:
15. Removing cluster1 from orgClusterResL;
16. Appending cluster1 to clusterResL;
17. end if
18. if orgClusterResL = {}:
19. break;
20. end while
21. Removing clusters from clusterResL with the number of cells <= allCellCount*0.01;
22. Obtaining the final clustering results (clusterResL).

3. **Supplementary Tables**

**3.1 Correlation matrices used for immune gene filtering**

**Table S1.1 Correlation matrix of immune genes in normal PBMC**

Pairwise Pearson correlation coefficients among immune genes used for initial gene set filtering in PBMC. The data can be accessed at: [https://github.com/347251369/Cross-Scale-Framework-for-Immune-System-Functional-Heterogeneity/blob/master/tables/Table S1.1 corMatrixPBMC.csv](https://github.com/347251369/Cross-Scale-Framework-for-Immune-System-Functional-Heterogeneity/blob/master/tables/Table%20S1.1%20corMatrixPBMC.csv)

**Table S1.2 Correlation matrix of immune genes in LUAD**

Pairwise Pearson correlation coefficients among immune genes used for initial gene set filtering in LUAD. The data can be accessed at: [https://github.com/347251369/Cross-Scale-Framework-for-Immune-System-Functional-Heterogeneity/blob/master/tables/Table S1.2 corMatrixLUAD.csv](https://github.com/347251369/Cross-Scale-Framework-for-Immune-System-Functional-Heterogeneity/blob/master/tables/Table%20S1.2%20corMatrixLUAD.csv)

**3.2 Bayesian gene-gene regulatory pairs**

**Table S2.1 High-confidence Bayesian regulatory gene pairs in normal PBMC**

Directed edges between immune genes inferred via Bayesian network learning in PBMC. The data can be accessed at: [https://github.com/347251369/Cross-Scale-Framework-for-Immune-System-Functional-Heterogeneity/blob/master/tables/Table S2.1 bayesGenePairPBMC.csv](https://github.com/347251369/Cross-Scale-Framework-for-Immune-System-Functional-Heterogeneity/blob/master/tables/Table%20S2.1%20bayesGenePairPBMC.csv)

**Table S2.2 High-confidence Bayesian regulatory gene pairs in LUAD**

Directed edges between immune genes inferred via Bayesian network learning in LUAD. The data can be accessed at: [https://github.com/347251369/Cross-Scale-Framework-for-Immune-System-Functional-Heterogeneity/blob/master/tables/Table S2.2 bayesGenePairLUAD.csv](https://github.com/347251369/Cross-Scale-Framework-for-Immune-System-Functional-Heterogeneity/blob/master/tables/Table%20S2.2%20bayesGenePairLUAD.csv)

**3.3 Network marker usage ratios across cell subsets**

**Table S3.1 ClusterRatio values of network markers in PBMC**

Fraction of cells within each PBMC cluster expressing the full marker triplet. The data can be accessed at: [https://github.com/347251369/Cross-Scale-Framework-for-Immune-System-Functional-Heterogeneity/blob/master/tables/Table S3.1 networkMarkerRatioPBMC.csv](https://github.com/347251369/Cross-Scale-Framework-for-Immune-System-Functional-Heterogeneity/blob/master/tables/Table%20S3.1%20networkMarkerRatioPBMC.csv)

**Table S3.2 ClusterRatio values of network markers in LUAD**

Fraction of cells within each LUAD cluster expressing the full marker triplet. The data can be accessed at: [https://github.com/347251369/Cross-Scale-Framework-for-Immune-System-Functional-Heterogeneity/blob/master/tables/Table S3.2 networkMarkerRatioLUAD.csv](https://github.com/347251369/Cross-Scale-Framework-for-Immune-System-Functional-Heterogeneity/blob/master/tables/Table%20S3.2%20networkMarkerRatioLUAD.csv)

**3.4 Summary statistics for immune cell subset biomarker systems**

**Table S4.1 Statistic results for immune cell subset network biomarker system of normal PBMC**

Summary table of the number of network markers, biomarkers, and clusters across immune subsets in normal PBMC. The data can be accessed at: [https://github.com/347251369/Cross-Scale-Framework-for-Immune-System-Functional-Heterogeneity/blob/master/tables/Table S4.1 Statistic results for immune cell subset network biomarker system of normal PBMC..csv](https://github.com/347251369/Cross-Scale-Framework-for-Immune-System-Functional-Heterogeneity/blob/master/tables/Table%20S4.1%20Statistic%20results%20for%20immune%20cell%20subset%20network%20biomarker%20system%20of%20normal%20PBMC..csv)

**Table S4.2 Statistic results for immune cell subset network biomarker system of LUAD**

Summary table of the number of network markers, biomarkers, and clusters across immune subsets in LUAD. The data can be accessed at: [https://github.com/347251369/Cross-Scale-Framework-for-Immune-System-Functional-Heterogeneity/blob/master/tables/Table S4.2 Statistic results for immune cell subset network biomarker system of LUAD..csv](https://github.com/347251369/Cross-Scale-Framework-for-Immune-System-Functional-Heterogeneity/blob/master/tables/Table%20S4.2%20Statistic%20results%20for%20immune%20cell%20subset%20network%20biomarker%20system%20of%20LUAD..csv)

**3.5 Raw single-cell RNA-seq data profiles**

**Table S5.1 Single-cell RNA-seq data of normal PBMC.**

The data is from the Institute of Thoracic Oncology and quality control has been completed. The data can be access at: <https://doi.org/10.5281/zenodo.15645068>

**Table S5.2 Single-cell RNA-seq data of LUAD.**

The data is from the Institute of Thoracic Oncology and quality control has been completed. The data can be access at: <https://doi.org/10.5281/zenodo.15645068>

**3.6 Raw bulk data profiles**

**Table S6.1 Bulk RNA-seq data of normal PBMC.**

Gene transcription and expression level data from normal PBMC, standardized by TPM. The data can be access at: [https://github.com/347251369/Cross-Scale-Framework-for-Immune-System-Functional-Heterogeneity/blob/master/tables/Table S6.1 Bulk RNA-seq data of normal PBMC.csv](https://github.com/347251369/Cross-Scale-Framework-for-Immune-System-Functional-Heterogeneity/blob/master/tables/Table%20S6.1%20Bulk%20RNA-seq%20data%20of%20normal%20PBMC.csv)

**Table S6.2 Bulk RNA-seq data of LUAD.**

Gene transcription and expression level data from LUAD, standardized by TPM. The data can be access at: [https://github.com/347251369/Cross-Scale-Framework-for-Immune-System-Functional-Heterogeneity/blob/master/tables/Table S6.2 Bulk RNA-seq data of LUAD.RData](https://github.com/347251369/Cross-Scale-Framework-for-Immune-System-Functional-Heterogeneity/blob/master/tables/Table%20S6.2%20Bulk%20RNA-seq%20data%20of%20LUAD.RData)

**3.7 Gene expression matrices from the normal PBMC and LUAD datasets**

**Table S7.1 Processed bulk expression matrix of normal PBMC.**

This data is processed expression data from normal PBMC. The data can be accessed at: [https://github.com/347251369/Cross-Scale-Framework-for-Immune-System-Functional-Heterogeneity/blob/master/tables/Table S7.1 bulkDataP.csv](https://github.com/347251369/Cross-Scale-Framework-for-Immune-System-Functional-Heterogeneity/blob/master/tables/Table%20S7.1%20bulkDataP.csv)

**Table S7.2 Processed bulk expression matrix of LUAD.**

This data is processed expression data from LUAD. The data can be accessed at: [https://github.com/347251369/Cross-Scale-Framework-for-Immune-System-Functional-Heterogeneity/blob/master/tables/Table S7.2 bulkDataL.RData](https://github.com/347251369/Cross-Scale-Framework-for-Immune-System-Functional-Heterogeneity/blob/master/tables/Table%20S7.2%20bulkDataL.RData)

**3.8 Immune-related pathway curation**

**Table S8 Immune-related pathways, including pathway introduction and genes.**

Curated immune-related pathways with functional annotations and gene membership information. The data can be accessed at: [https://github.com/347251369/Cross-Scale-Framework-for-Immune-System-Functional-Heterogeneity/blob/master/tables/Table S8 immunePathways.csv](https://github.com/347251369/Cross-Scale-Framework-for-Immune-System-Functional-Heterogeneity/blob/master/tables/Table%20S8%20immunePathways.csv)

**3.9 Immune gene set definition**

**Table S9** **Immune genes from immune Pathways**

A comprehensive list of immune-related genes extracted from curated pathways, including pathway names, functional annotations, and gene members. The data can be accessed at: [https://github.com/347251369/Cross-Scale-Framework-for-Immune-System-Functional-Heterogeneity/blob/master/tables/Table S9 immuneGeneSet.csv](https://github.com/347251369/Cross-Scale-Framework-for-Immune-System-Functional-Heterogeneity/blob/master/tables/Table%20S9%20immuneGeneSet.csv)

**3.10 Immune gene set bulk RNA-seq data**

**Table S10.1** **Immune gene set bulk RNA-seq data of normal PBMC**

Expression matrix of immune-related genes extracted from the bulk RNA-seq dataset of normal PBMC. The data can be accessed at: [https://github.com/347251369/Cross-Scale-Framework-for-Immune-System-Functional-Heterogeneity/blob/master/tables/Table S10.1 immuneGeneSetP.csv](https://github.com/347251369/Cross-Scale-Framework-for-Immune-System-Functional-Heterogeneity/blob/master/tables/Table%20S10.1%20immuneGeneSetP.csv)

**Table S10.2 Immune gene set bulk RNA-seq data of LUAD**

Expression matrix of immune-related genes extracted from the bulk RNA-seq dataset of LUAD. The data can be accessed at: [https://github.com/347251369/Cross-Scale-Framework-for-Immune-System-Functional-Heterogeneity/blob/master/tables/Table S10.2 immuneGeneSetL.csv](https://github.com/347251369/Cross-Scale-Framework-for-Immune-System-Functional-Heterogeneity/blob/master/tables/Table%20S10.2%20immuneGeneSetL.csv)

**3.11 results of Correlation analysis**

**Table S11.1** **Correlation analysis results of normal PBMC**

The data can be accessed at: [https://github.com/347251369/Cross-Scale-Framework-for-Immune-System-Functional-Heterogeneity/blob/master/tables/Table S11.1 highImmuneGeneSetP.csv](https://github.com/347251369/Cross-Scale-Framework-for-Immune-System-Functional-Heterogeneity/blob/master/tables/Table%20S11.1%20highImmuneGeneSetP.csv)

**Table S11.2 Correlation analysis results of LUAD**

The data can be accessed at: [https://github.com/347251369/Cross-Scale-Framework-for-Immune-System-Functional-Heterogeneity/blob/master/tables/Table S11.2 highImmuneGeneSetL.csv](https://github.com/347251369/Cross-Scale-Framework-for-Immune-System-Functional-Heterogeneity/blob/master/tables/Table%20S11.2%20highImmuneGeneSetL.csv)

**3.12 Results of Bayesian network structure analysis**

**Table S12.1** **Bayesian network structure analysis results of normal PBMC**

The data can be accessed at: [https://github.com/347251369/Cross-Scale-Framework-for-Immune-System-Functional-Heterogeneity/blob/master/tables/Table S12.1 causalityNetworkP.csv](https://github.com/347251369/Cross-Scale-Framework-for-Immune-System-Functional-Heterogeneity/blob/master/tables/Table%20S12.1%20causalityNetworkP.csv)

**Table S12.2 Bayesian network structure analysis results of LUAD**

The data can be accessed at: [https://github.com/347251369/Cross-Scale-Framework-for-Immune-System-Functional-Heterogeneity/blob/master/tables/Table S12.2 causalityNetworkL.csv](https://github.com/347251369/Cross-Scale-Framework-for-Immune-System-Functional-Heterogeneity/blob/master/tables/Table%20S12.2%20causalityNetworkL.csv)

**3.13 Unsupervised clustering and immune cell type annotation**

**Table S13.1 Unsupervised clustering and immune cell type annotation results** **of normal PBMC**

Clustering assignments and manually curated immune cell type annotations for single-cell RNA-seq data of normal PBMC. The data can be accessed at: [https://github.com/347251369/Cross-Scale-Framework-for-Immune-System-Functional-Heterogeneity/blob/master/tables/Table S13.1 annoDataP.csv](https://github.com/347251369/Cross-Scale-Framework-for-Immune-System-Functional-Heterogeneity/blob/master/tables/Table%20S13.1%20annoDataP.csv)

**Table S13.2 Unsupervised clustering and immune cell type annotation results of LUAD**

Clustering assignments and manually curated immune cell type annotations for single-cell RNA-seq data of LUAD. The data can be accessed at: [https://github.com/347251369/Cross-Scale-Framework-for-Immune-System-Functional-Heterogeneity/blob/master/tables/Table S13.2 annoDataL.csv](https://github.com/347251369/Cross-Scale-Framework-for-Immune-System-Functional-Heterogeneity/blob/master/tables/Table%20S13.2%20annoDataL.csv)

**3.14 Candidate network marker identification**

**Table S14.1 Candidate network markers of normal PBMC**

Preliminary list of network marker candidates identified from the normal PBMC Bayesian network-derived structure. The data can be accessed at: [https://github.com/347251369/Cross-Scale-Framework-for-Immune-System-Functional-Heterogeneity/blob/master/tables/Table S14.1 candNetworkMarkerP.csv](https://github.com/347251369/Cross-Scale-Framework-for-Immune-System-Functional-Heterogeneity/blob/master/tables/Table%20S14.1%20candNetworkMarkerP.csv)

**Table S14.2 Candidate network markers of LUAD**

Preliminary list of network marker candidates identified from the LUAD Bayesian network-derived structure The data can be accessed at: [https://github.com/347251369/Cross-Scale-Framework-for-Immune-System-Functional-Heterogeneity/blob/master/tables/Table S14.2 candNetworkMarkerL.csv](https://github.com/347251369/Cross-Scale-Framework-for-Immune-System-Functional-Heterogeneity/blob/master/tables/Table%20S14.2%20candNetworkMarkerL.csv)

**3.15 Candidate network marker identification**

**Table S15.1 Candidate network markers of normal PBMC**

Preliminary list of network marker candidates identified from the normal PBMC causality network. The data can be accessed at: [https://github.com/347251369/Cross-Scale-Framework-for-Immune-System-Functional-Heterogeneity/blob/master/tables/Table S15.1 networkMarkerP.xlsx](https://github.com/347251369/Cross-Scale-Framework-for-Immune-System-Functional-Heterogeneity/blob/master/tables/Table%20S15.1%20networkMarkerP.xlsx)

**Table S15.2 Candidate network markers of LUAD**

Preliminary list of network marker candidates identified from the LUAD causality network The data can be accessed at: [https://github.com/347251369/Cross-Scale-Framework-for-Immune-System-Functional-Heterogeneity/blob/master/tables/Table S15.2 networkMarkerL.xlsx](https://github.com/347251369/Cross-Scale-Framework-for-Immune-System-Functional-Heterogeneity/blob/master/tables/Table%20S15.2%20networkMarkerL.xlsx)

**3.16 Cell clustering results across immune subsets**

**Table S16.1 Cell clusters results for each cell subset of normal PBMC**

Final clustering assignments for immune lineages in normal PBMC, generated using structure-guided marker expression. The data can be accessed at: [https://github.com/347251369/Cross-Scale-Framework-for-Immune-System-Functional-Heterogeneity/tree/master/tables/Table S16.1 Network biomarker system of normal PBMC](https://github.com/347251369/Cross-Scale-Framework-for-Immune-System-Functional-Heterogeneity/tree/master/tables/Table%20S16.1%20Network%20biomarker%20system%20of%20normal%20PBMC)

**Table S16.2. Cell clusters results for each cell subset of LUAD**

Final clustering assignments for immune lineages in LUAD, incorporating iterative refinement of network biomarker sets. The data can be accessed at: [https://github.com/347251369/Cross-Scale-Framework-for-Immune-System-Functional-Heterogeneity/tree/master/tables/Table S16.2 Network biomarker system of LUAD](https://github.com/347251369/Cross-Scale-Framework-for-Immune-System-Functional-Heterogeneity/tree/master/tables/Table%20S16.2%20Network%20biomarker%20system%20of%20LUAD)

**3.17 Cell cluster visualization**

**Table S17.1 Cell cluster visualization results for each immune cell subset in normal PBMC**

UMAP plots showing the distribution of final structure-defined clusters within each immune cell lineage from normal PBMC. The data can be accessed at: [https://github.com/347251369/Cross-Scale-Framework-for-Immune-System-Functional-Heterogeneity/blob/master/tables/Table S17.1 cellClusterVisP.xlsx](https://github.com/347251369/Cross-Scale-Framework-for-Immune-System-Functional-Heterogeneity/blob/master/tables/Table%20S17.1%20cellClusterVisP.xlsx)

**Table S17.2 Cell cluster visualization results for each immune cell subset in LUAD**

UMAP plots showing the distribution of final structure-defined clusters within each immune cell lineage from LUAD. The data can be accessed at: [https://github.com/347251369/Cross-Scale-Framework-for-Immune-System-Functional-Heterogeneity/blob/master/tables/Table S17.2 cellClusterVisL.xlsx](https://github.com/347251369/Cross-Scale-Framework-for-Immune-System-Functional-Heterogeneity/blob/master/tables/Table%20S17.2%20cellClusterVisL.xlsx)

**3.18 Functional definition of immune subclusters**

**Table S18.1 Cell cluster definition results for cell clusters from each immune cell subset in normal PBMC**

Differential expression and GO enrichment results for each immune subcluster identified in normal PBMC. The data can be accessed at: [https://github.com/347251369/Cross-Scale-Framework-for-Immune-System-Functional-Heterogeneity/blob/master/tables/Table S18.1 cellClusterDeP.xlsx](https://github.com/347251369/Cross-Scale-Framework-for-Immune-System-Functional-Heterogeneity/blob/master/tables/Table%20S18.1%20cellClusterDeP.xlsx)

**Table S18.2 Cell cluster definition results for cell clusters from each immune cell subset in LUAD**

Differential expression and GO enrichment results for each immune subcluster identified in normal PBMC. The data can be accessed at: [https://github.com/347251369/Cross-Scale-Framework-for-Immune-System-Functional-Heterogeneity/blob/master/tables/Table S18.2 cellClusterDeL.xlsx](https://github.com/347251369/Cross-Scale-Framework-for-Immune-System-Functional-Heterogeneity/blob/master/tables/Table%20S18.2%20cellClusterDeL.xlsx)

**3.19 Raw Spatial transcriptome data from the Stereo-seq platform**

**Table S19 Raw spatial transcriptome data from the stereo-seq platform**

Original unprocessed transcript count matrices from spatially barcoded tissue sections. The data can be accessed at: <https://doi.org/10.5281/zenodo.15645068>

**3.20 Processed spatial transcriptome data from the stereo-seq platform**

**Table S20 Processed spatial transcriptome data from the stereo-seq platform**

Spatial transcriptome data after quality control, filtering, and normalization. The data can be accessed at: <https://doi.org/10.5281/zenodo.15645068>

**3.21 Unsupervised clustering and annotation of spatial transcriptome data**

**Table S21 Unsupervised clustering and immune cell type annotation results of stereo-seq data**

Cluster assignments and cell type annotations for spatial transcriptome data in LUAD tissue sections. The data can be accessed at: [https://github.com/347251369/Cross-Scale-Framework-for-Immune-System-Functional-Heterogeneity/blob/master/tables/Table S21 annoStereoData.xlsx](https://github.com/347251369/Cross-Scale-Framework-for-Immune-System-Functional-Heterogeneity/blob/master/tables/Table%20S21%20annoStereoData.xlsx)

**3.22 Network-derived associations between network biomarkers**

**Table S22 Network-derived associations between network biomarkers**

Biomarker pairs linked by Bayesian network-derived associations, together with their corresponding confidence values. The data can be accessed at: [https://github.com/347251369/Cross-Scale-Framework-for-Immune-System-Functional-Heterogeneity/blob/master/tables/Table S22 clusterTOcluster.csv](https://github.com/347251369/Cross-Scale-Framework-for-Immune-System-Functional-Heterogeneity/blob/master/tables/Table%20S22%20clusterTOcluster.csv)

**3.23 Spatial analysis of network-associated biomarker-defined clusters**

**Table S23 Analysis results of network-derived associations between network biomarkers and the spatial distribution of cells**

Pairwise Euclidean distances between biomarker-defined clusters grouped by the presence or absence of Bayesian network-derived associations. The data can be accessed at: [https://github.com/347251369/Cross-Scale-Framework-for-Immune-System-Functional-Heterogeneity/blob/master/tables/Table S23 cellSpatialDist.xlsx](https://github.com/347251369/Cross-Scale-Framework-for-Immune-System-Functional-Heterogeneity/blob/master/tables/Table%20S23%20cellSpatialDist.xlsx)

**3.24 Literature-anchored functional annotation of causal regulatory markers**

**Table S24 Functional annotation of causal regulatory markers across PBMC and LUAD subsets based on literature evidence**

Manually curated literature references supporting the immune-related functions of causal regulatory markers identified in PBMC and LUAD. Each marker is annotated with reported functions, associated immune processes, and PubMed IDs for traceability. The data can be accessed at https://github.com/347251369/Cross-Scale-Framework-for-Immune-System-Functional-Heterogeneity/blob/master/tables/Table_S24_Literature-anchored_functional_annotation.xlsx

4. **Supplementary Codes**

The codes for Regulatory Structure-Based Cell State Identification and Spatial Coherence Analysis are available at: <https://github.com/347251369/Cross-Scale-Framework-for-Immune-System-Functional-Heterogeneity/tree/master/code>
